# A Satellite dsRNA Attenuates the Induction of Helper Virus-Mediated Symptoms in *Aspergillus flavus*

**DOI:** 10.3389/fmicb.2022.895844

**Published:** 2022-05-31

**Authors:** Yinhui Jiang, Bi Yang, Xiang Liu, Xun Tian, Qinrong Wang, Bi Wang, Qifang Zhang, Wenfeng Yu, Xiaolan Qi, Yanping Jiang, Tom Hsiang

**Affiliations:** ^1^Key Laboratory of Endemic and Ethnic Diseases, Ministry of Education, Guizhou Medical University, Guiyang, China; ^2^Key Laboratory of Medical Molecular Biology, Guizhou Medical University, Guiyang, China; ^3^Department of Dermatology, The Affiliated Hospital, Guizhou Medical University, Guiyang, China; ^4^School of Environmental Sciences, University of Guelph, Guelph, ON, Canada

**Keywords:** *Aspergillus flavus*, Aspergillus flavus partitivirus 1, a satellite dsRNA, virus therapy, transcriptional analysis

## Abstract

*Aspergillus flavus* is an important fungal pathogen of animals and plants. Previously, we reported a novel partitivirus, Aspergillus flavus partitivirus 1 (AfPV1), infecting *A. flavus*. In this study, we obtained a small double-stranded (ds) RNA segment (734 bp), which is a satellite RNA of the helper virus, AfPV1. The presence of AfPV1 altered the colony morphology, decreased the number of conidiophores, created significantly larger vacuoles, and caused more sensitivity to osmotic, oxidative, and UV stresses in *A. flavus*, but the small RNA segment could attenuate the above symptoms caused by the helper virus AfPV1 in *A. flavus*. Moreover, AfPV1 infection reduced the pathogenicity of *A. flavus* in corn (*Zea mays*), honeycomb moth (*Galleria mellonella*), mice (*Mus musculus*), and the adhesion of conidia to host epithelial cells, and increased conidial death by macrophages. However, the small RNA segment could also attenuate the above symptoms caused by the helper virus AfPV1 in *A. flavus*, perhaps by reducing the genomic accumulation of the helper virus AfPV1 in *A. flavus*. We used this model to investigate transcriptional genes regulated by AfPV1 and the small RNA segment in *A. flavus*, and their role in generating different phenotypes. We found that the pathways of the genes regulated by AfPV1 in its host were similar to those of retroviral viruses. Therefore, some pathways may be of benefit to non-retroviral viral integration or endogenization into the genomes of its host. Moreover, some potential antiviral substances were also found in *A. flavus* using this system.

## Introduction

Mycoviruses are widely distributed in fungi, and have single-stranded (ss), double-stranded (ds) RNA or DNA genomes (Yu et al., 2010; Liu et al., 2014). Currently, the recognized mycoviruses are comprised of seven families: *Totiviridae*, *Partitiviridae*, *Megabirnaviridae*, *Chrysoviridae*, *Quadriviridae*, *Endornaviridae*, and *Reoviridae* (Ghabrial et al., 2015). Unlike viruses infecting plants and animals, the transmission of mycoviruses often occurs vertically by sporulation or horizontally via hyphal anastomosis (Pearson et al., 2009), with rare extracellular transmission (Yu et al., 2010, 2013). Most mycoviruses are typically latent infections, but some do cause obvious symptoms such as debilitated virulence, slow growth rate, and poor sporulation of their host (Ghabrial and Suzuki, 2009; Xie and Jiang, 2014). Considering these effects, some mycoviruses may offer great potential in the management of fungal diseases (Nuss, 2005; Chiba et al., 2009; Yu et al., 2013; Xie and Jiang, 2014).

Recently, it has been reported that some mycoviruses additionally harbor satellite RNA segments (Liu et al., 2015; Shah et al., 2015). Most satellite segments are classified as satellite viruses and satellite nucleic acids whether DNA or RNA (Gnanasekaran and Chakraborty, 2018). Satellite viruses encode structural proteins which are essential for the encapsidation of their own genome, and distinct from the main mycovirus also called the helper virus (Krupovic et al., 2016; Gnanasekaran and Chakraborty, 2018). There are no structural proteins encoded by satellite segments, which are instead encapsidated by helper virus-encoded capsid proteins (CPs) (Gnanasekaran and Chakraborty, 2018). However, the molecular mechanisms of interactions among hosts, satellites, and helper viruses remain largely unexplored. It is widely accepted that satellite segments are dependent on their helper viruses for replication and encapsidation, as they do not encode any RNA/DNA polymerases (Wang and Smith, 2015). Despite this dependence, satellite segments rarely share sequence similarity with helper viruses (Gnanasekaran and Chakraborty, 2018). For plant viruses, satellite segments are able to exacerbate or attenuate the symptoms in their hosts (Kaper and Waterworth, 1977; Waterworth et al., 1979; Wang and Smith, 2015). Perhaps satellite segments alter (impede or increase) the genomic accumulation of the helper virus in the infected host cell (Gnanasekaran and Chakraborty, 2018). But, very few cause-effect relationships between satellite segments and helper viruses have been established for mycovirus infections, and there is only a report in the model fungus *Saccharomyces cerevisiae* (Gnanasekaran and Chakraborty, 2018). Some *S. cerevisiae* strains are infected by a dsRNA virus of the *Totiviridae* family and a satellite dsRNA segment. The satellite dsRNA segment produces a killer toxin that lyses susceptible neighboring *S. cerevisiae* cells, but the virus-containing *S. cerevisiae* strain is immune to this toxin (Schmitt and Tipper, 1990; Rodríguez-Cousiño et al., 2011).

Although mycoviruses are widespread among fungi, the interactions between the mycovirus and its host are not well understood. RNA silencing is one of antiviral defense mechanisms in the fungus *Cryphonectria parasitica* (Segers et al., 2007). Mutations of the RNA silencing pathway in *C. parasitica* increases the susceptibility to virus infections in *C. parasitica*, and the viral protein p29 is considered a suppressor of RNA silencing-mediated viral defense in the fungus (Xuemin et al., 2008). High throughput RNA-seq is a powerful tool to provide comprehensive information on interactions between mycoviruses and their hosts (Son et al., 2015; Stark et al., 2019). The transcriptome of fungi containing mycoviruses can reveal the mechanisms of mycovirus-mediated symptoms in fungi. However, there are only a few studies investigating mycovirus-mediated transcriptional or translational changes in fungi, including mycoviruses associated with *Aspergillus fumigatus*, *C. parasitica*, *Rosellinia necatrix*, *Sclerotinia sclerotiorum*, or *Fusarium graminearum* (Lee et al., 2014; Lee Marzano et al., 2018; Shimizu et al., 2018; Takahashi-Nakaguchi et al., 2019; Chun et al., 2020). Many biological processes are involved in virus-host interactions, but in most cases the exact nature of the gene expression and their pathways modified by mycoviruses is unknown (Kotta-Loizou, 2021).

*Aspergillus flavus* is a ubiquitous fungus, which can contaminate many crops and cause large economic losses (Amaike and Keller, 2011; Almeida et al., 2019). Moreover, *A. flavus*-contaminated food can be dangerous due to aflatoxin, which is a natural hepatocarcinogenic secondary metabolite causing acute aflatoxicosis and hepatocellular carcinoma (Turner et al., 2003; Kensler et al., 2011; Lohmar et al., 2019). *A. flavus* is also an opportunistic pathogen causing aspergillosis diseases in immunocompromised humans (Almeida et al., 2019). Currently, only antimicrobial agents are available to treat aspergillosis, but antifungal antibiotics often have toxicity to humans and may easily lead to fungicide resistance (Arendrup, 2014; Paul et al., 2015). Therefore, an urgent need exists for new therapeutic strategies to control *A. flavus* infections. Mycoviruses have been considered biocontrol agents in phytopathogenic fungi such as *C. parasitica*, *S. sclerotiorum*, *Helminthosporium victoriae*, and *Botrytis cinerea* (Van Alfen et al., 1975; Choi and Nuss, 1992; Xie and Jiang, 2014; Jia et al., 2021). With increasing reports of novel mycoviruses selectively infecting fungi which can infect humans, mycoviruses as therapeutic strategies in human fungal infections show increasing prominence (Van De Sande et al., 2010; Bhatti et al., 2011; Özkan and Coutts, 2015). Furthermore, mycoviruses have also been found in *A. flavus*, so it is possible that a mycovirus associated with hypovirulence may be used as a novel therapeutic solution for *A. flavus* infections (Kotta-Loizou and Coutts, 2017; Van De Sande and Vonk, 2019).

In a previous study, we reported a novel partitivirus, Aspergillus flavus partitivirus 1 (AfPV1), causing debilitation of its host, *A. flavus* (Jiang et al., 2019). Recently, we found a small dsRNA (734 bp) that may be a satellite RNA of helper virus AfPV1 (Gnanasekaran and Chakraborty, 2018), and the small dsRNA showed conserved nucleotides (AAACUUU) at the 5′-terminus with AfPV1 (Jiang et al., 2019). This report presents the first example of a partitivirus harboring a satellite RNA which can affect the induction of helper virus-mediated symptoms in its host, *A. flavus.* The modulated gene expression of *A. flavus* in response to infection by AfPV1 and the SatRNA was further analyzed by transcriptome profiling.

## Materials and Methods

### Fungal Isolates and Growth Conditions

A single-spore isolate *A. flavus* ZD1.22-10-9 which harbors AfPV1 and satellite RNA was isolated from the air of a hospital room in the Guizhou Province People’s Hospital, Guiyang, Guizhou, China. Another single-spore isolate, *A. flavus* LD-3-8, harboring AfPV1, was isolated from the Affiliated Hospital of Guizhou Medical University, also in Guiyang. Isolates LD-F, LD_F1, and LD_F1-b were also previously obtained and investigated (Jiang et al., 2019). A virus-free isolate, LD-F, was obtained from isolate LD-3-8 by single sporing. Isolate LD-F1 was derived from LD-F by labeling with a pyrithiamine resistance (*ptr*) gene using PEG-mediated methods (Chang et al., 2010; Jiang et al., 2019). Isolate LD-F1-b containing AfPV1, was obtained by transferring AfPV1 from isolate LD-3-8 (donor) to the virus-free isolate LD-F1 (recipient) (Jiang et al., 2019). Isolate LD-F1-a containing AfPV1 and satellite RNA, was obtained by transferring AfPV1 and satellite RNA from isolate ZD1.22-10-9 (donor) to the virus-free isolate LD-F1 (recipient) (Figures 2A,B; Jiang et al., 2019). All *A. flavus* isolates were cultured on potato dextrose agar (PDA) plates at 30°C and stored on PDA slants at 4°C.

### Extraction and Purification of dsRNAs From Mycelia and Viral Particles

Isolates of *A. flavus* were cultured on sterilized cellophane films placed on PDA plates and incubated at 30°C in the dark for 5 days. Mycelia of each isolate were harvested and stored at –80°C until use. DsRNA elements from mycelia were extracted and purified using CF-11 cellulose (Sigma, St. Louis, MO, United States) column chromatography as previously described (Morris and Dodds, 1979). Extraction and purification of viral particles from *A. flavus* followed previously described methods (Yu et al., 2010; Jiang et al., 2019). The isolates were cultured at 30°C for 5 days on sterilized cellophane films placed on PDA. Approximately 25 g mycelia were ground in liquid nitrogen and mixed with four volumes of 0.01 M phosphate buffer saline (PBS, pH 7.4), and then the mixture was gently shaken on ice for 30 min. The mixture was separated with high-speed centrifugation (10,000 × *g* for 30 min). The supernatant was then subjected to ultracentrifugation at 120,000 × *g* for 2 h under 4°C to precipitate the virus particles in the supernatant. The supernatant containing the virus particles was then placed in a centrifuge tube containing a sucrose density gradient (10–40%), and centrifuged at 70,000 × *g* for 3 h at 4°C. The gradient fraction which could contain virus-like particles was diluted with PBS and ultracentrifuged at 120,000 × *g* for 2 h at 4°C. Five separated fractions were individually measured for the presence of the virus particles by dsRNA detection using agarose gel electrophoresis and then viewed on an UV *trans*-illuminator after staining with ethidium bromide.

### cDNA Cloning, Sequence Analysis, and Northern Blot Analysis

The individual dsRNA segments were cut from agarose gel for cDNA synthesis and molecular cloning of dsRNAs using random PCR amplification. Sequencing of the cDNAs were done using the random-primer (5′-CGATCGATCATGATGCAATGCNNNNNN-3′) amplification procedures described in our previous studies (Jiang et al., 2019). The end of each dsRNA segment sequence was amplified as described previously (Potgieter et al., 2009). The expected PCR products were cloned into the pMD18-T vector (TaKaRa, Dalian, China) and sequenced at Sangon Biotech (Shanghai, China). The full-length cDNA sequence of each dsRNA4 was assembled and aligned with DNAMAN 7.0 (Lynnon Biosoft, Quebec, QC Canada).

Northern blot analysis was performed following the previous study with minor modifications (Jiang et al., 2015). The dsRNA segments were transferred to nylon membrane (GE Healthcare, Buckinghamshire, United Kingdom) by capillary action. Hybridization was performed using a DIG High Prime DNA Labeling and Detection Starter Kit II (Roche, Mannheim, Germany), and the chemiluminescent signals of the probe hybrids were detected using a chemiluminescent substrate, CSPD (Roche, Mannheim, Germany). The specific DNA probes were amplified by primer pairs based on the genome sequence of dsRNA4 (satellite RNA) and 3′-terminus genome sequences of dsRNA1, dsRNA2, and dsRNA3 (AfPV1), which were non-conserved regions. The sequences of probe-dsRNA1, probe-dsRNA2, probe-dsRNA3, and probe-dsRNA4 are shown in Supplementary Table S1.

### Horizontal Transmission of dsRNAs

dsRNAs-infected isolates (donor) were individually co-cultured with pyrithiamine resistance isolate LD-F1 (recipient) on a CZ plate (9 cm in diameter) for 10 days at 30°C. Mycelial agar plugs from the margin areas of isolate LD-F1 were transferred to a fresh CZ plate containing 0.2 μg/mL pyrithiamine and subcultured at 30°C through five serial transfers onto pyrithiamine-amended media to allow only labeled isolates to grow, and then transferred to fresh PDA and CZ plates without pyrithiamine. The horizontal transmission of dsRNA was assayed by dsRNA extraction and RT-PCR amplification. The accumulation of dsRNAs was also determined by total RNA extraction and quantitative reverse transcriptase PCR (qRT-PCR) amplification. The primers for specific detection of each dsRNA are shown in Supplementary Table S2.

### Sporulation

Sporulation was assessed for cultures on PDA plates (9 cm in diameter) incubated at 30°C in the dark for 6 days (five replicate plates for each isolate). The conidia were harvested in 2 mL saline buffer, and spore numbers were estimated using a hemocytometer.

### Stress Assay

Measurements of stress tolerance followed previous methods with slight modifications (Takahashi-Nakaguchi et al., 2019; Takahashi-Nakaguchi et al., 2020). Conidia of *A flavus* (1 × 10^6^ spores/mL) were placed onto yeast-glucose minimal medium (YGM: 0.1% yeast cream, 1% glucose, and 1.5% agar) supplemented with the following agents: cell wall stress agents Congo red (CR), hyperosmotic stress mediator sodium chloride (NaCl, 1 M), or oxidative stress agent hydrogen peroxide (H_2_O_2_). The inoculated plates were incubated at 30°C in darkness for 5 days. Colony diameters were measured every day, each isolate was cultured on five replicate plates, and each experiment was repeated three times. The percent radial growth inhibition (RGI) was determined as follows: (radial colony diameters without any stress - radial colony diameters with stress)/radial colony diameters without any stress × 100. To measure growth under UV stress conditions (ultraviolet light, 60 w/cm^2^ for 30, 60, 90, 120, and 150 s), 1000 conidia (1 ml of 10^3^ conidia/ml spore suspension streaked onto 9 cm diameter plates) were incubated on Sabouraud dextrose agar (SDA, 1% peptone, 4% glucose, 2% agar) at 37°C, and the number of visible colonies was assessed. The percent inhibition ratio under UV stress was calculated as follows: (colony number on SDA without UV – colony number on SDA without UV)/colony number on SDA without UV × 100.

### Mortality Assay by Macrophages

This procedure was based on a previous study with slight modifications (Lau et al., 2018). Murine RAW264.7 macrophages were transferred to 6-well plates at a density of 4 × 10^5^ cells/well and cultured in a complete medium (containing 10% Foetal Bovine Serum, FBS) at 37°C in 5% CO_2_ for 12 h. Then, the medium was removed and cells were washed twice with PBS, and 2 mL fresh medium was added to each well. Conidia (10^7^ spores) were added to each well, and the plates were incubated at 37°C under 5% CO_2_. After 2 h of incubation, the medium was discarded and cells were washed with PBS five times to eliminate conidia which had not adhered to RAW 264.7 macrophages. Then 2 mL fresh medium was added to each well and the plates were incubated for 0, 1, and 3 h to permit phagocytosis. After that, the macrophages were lysed with 0.2% Triton-X100 for 20 min at room temperature. Finally, the macrophages containing conidia were inoculated onto Sharburg medium at 30°C after gradient dilutions (0, 5x, and 10x), and the number of colonies was calculated. For the 0 h treatment, the number of colonies represented low exposure to macrophages. For 1 and 3 h incubation times, the number of colonies represented conidial survival after exposure to phagocytosis by macrophages. The conidial mortality (%) was determined as number of colonies at 0 h minus the number of colonies at different time exposures all divided by colony number at 0 h exposure.

### Adherence Assay

This followed a previously described method (Takahashi-Nakaguchi et al., 2020). The type II human pneumocyte cell line A549 was maintained in RPMI 1640 medium (Gibco) containing 10% fetal bovine serum (FBS, ExcCell Bio), 10000 units/mL penicillin, and 10,000 ug/mL streptomycin (Gibco) at 37°C in a humidified 5% CO_2_ incubator. The cells were plated at 10^5^ cells/well in 6-well culture plates and grown to confluence. Each well was overlaid with RPMI 1640 medium containing 100 conidia and cultured at 37°C under 5% CO_2_. The control was overlaid with RPMI 1640 medium without conidia. After 2 h incubation, each well was washed with PBS 3 times, and then overlaid with Sabouraud dextrose agar. After 26 h incubation at 37°C, colonies in each well were counted. Conidial adherence levels were shown as the percentage ± SD of the number of colonies in each well divided by the original conidial number.

### Pathogenicity Testing

With slight modifications, the procedures for corn kernel inoculation followed previous work (Yang et al., 2017, 2019). Fresh corn kernels of similar size and shape were placed in conical flasks containing 20 mL of different spores concentrations (0, 10^3^, 10^4^, 10^5^, 10^6^, 10^7^, and 10^8^ conidia/mL) of *A. flavus*, and shaken at 80 rpm for 30 min. And then the corn kernels were placed in petri plates lined with moist absorbent cotton and incubated at 30°C for 7 days. Ten corn kernels were sampled for each treatment. The infected seeds were fully washed with 10 mL PBS containing 0.05% Tween 80, and then spore count was assessed. There were five replicates per isolate by concentration combination.

Final instar larvae of the honeycomb moth (*G. mellonella*) were obtained from Tianjin Huiyude Biotechnology Co., Ltd. (Tianjin, China). In order to observe pigmentation changes clearly, cream-colored larvae weighing approximately 0.2 g each were selected and injected with 10 μl *A. flavus* spore suspension at various concentrations (10, 10^2^, 10^3^, 10^4^, 10^5^, and 10^6^ spores/larva) through the last left proleg into the hemocoel using a microsyringe (Özkan and Coutts, 2015). Ten larvae were used for each experiment, and each experiment was independently repeated three times. Control and check treatments were untouched larvae (UTC), pierced larvae (PC), and PBS-injected larvae (PBS). For survival rate assays, all the larvae were incubated in Petri dishes in the dark at 37°C for 5 days, and their times of death (no response to touch) were recorded daily together with notes on any melanization (an indicator of fungal growth and an active immune response). Survival curves were plotted using the GraphPad Prism 6.0 program and the statistical significance were determined using Log rank tests at *p* = 0.05. For histopathological examination, each group of larvae at 48 h post-inoculation were placed in 10% paraformaldehyde and then stained with Hematoxylin and eosin (HE) or Grocott’s methenamine silver (GMS).

Female ICR mice (specific pathogen-free, 6-weeks old, weighing from 16 to 18 g each) were purchased from the Guizhou Laboratory Animal Engineering Technology Center (Guiyang, China). The procedures with slight modifications were described previously (Jiang et al., 2019; Takahashi-Nakaguchi et al., 2020). The mice were allowed free access to food, and in order to prevent bacterial infections, the drinking water was amended with 100 mg/L cephalosporin. A dose of 250 mg/kg cyclophosphamide (Sigma, St Louis, MO, United States) was injected to the mice intraperitoneally on days −3, −1, and +2 before inoculation to cause immunosuppression. On day 0, inocula containing 10^4^, 10^6^, or 10^8^ CFU/mL conidia were prepared from *A. flavus* isolates in saline buffer. For the intratracheal injection experimental group, each immunosuppressed mouse was anesthetized by injection of chloral hydrate and intravenously inoculated with 50 μL of each conidial suspension via intranasal instillation. For the intravenous injection experimental group, 40 μl of each conidial suspension was intravenously inoculated into each immunosuppressive mouse via the lateral tail vein. Twenty mice per group were used, and control experiments were composed of untreated mice (UTC) and saline buffer injected immunosuppressive mice (Mock). Morbidity and viability were monitored for up to 14 days. To determine *A. flavus* burden, at least five mice from each group were sacrificed on 1, 3, 5, and 7 days after inoculation, and then the lung, liver, and kidney tissues were weighed, homogenized, and serially diluted onto SDA plus streptomycin and pyrithiamine at 30°C. The number of colonies per gram of the tissue (CFU/g) was detected after 24 h. The fungal burden was assessed as Log 10 CFU/g of tissue, and each tissue type was repeated at least three times. For histopathological examination, the tissue of each group of mice at 72 h post-inoculation were placed into 10% paraformaldehyde and then stained with HE or GMS separately.

### Transcriptional Analysis and qRT-PCR Amplification

For RNA-seq analysis, mycelia of isolates LD-F1, LD-F1-b and LD-F1-a were placed on cellophane membranes overlaid onto PDA and grown at 30°C for 5 days. The mycelia of each isolate were harvested, and total RNA was extracted using the TRIzol reagent following manufacturer’s instructions (Invitrogen, Grand Island, Germany). The RNA samples were sequenced by Novogene, Ltd (Beijing, China) on the Illumina HiSeq 2000 platform, and each isolate was repeated in triplicate. Sequence analyses by Novogene were as follows: (1) the raw reads were filtered into clean reads by removing low quality bases and contaminating sequences, and subsequently mapped to a reference genome of *A. flavus* NRRL 3357 from the National Center for Biotechnology Information (NCBI^1^); and (2) based on *q*-value less than 0.05, differentially expressed genes (DEGs) were determined by calculating the RPKM (Reads Per Kilobase per Million mapped reads). The Database for Annotation, Visualization, and Integrated Discovery (DAVID^2^) was used to analyze the functional enrichment of InterPro domains, gene ontology (GO) and Kyoto encyclopedia of genes and genomes (KEGG) pathway. qRT-PCR was also done to confirmed the differential expressed genes, with primers used for RT-PCR and qPCR analyses listed in Supplementary Table S2.

## Results

### dsRNA Elements in *Aspergillus flavus* Isolate ZD1.22-10-9

**FIGURE 1:**
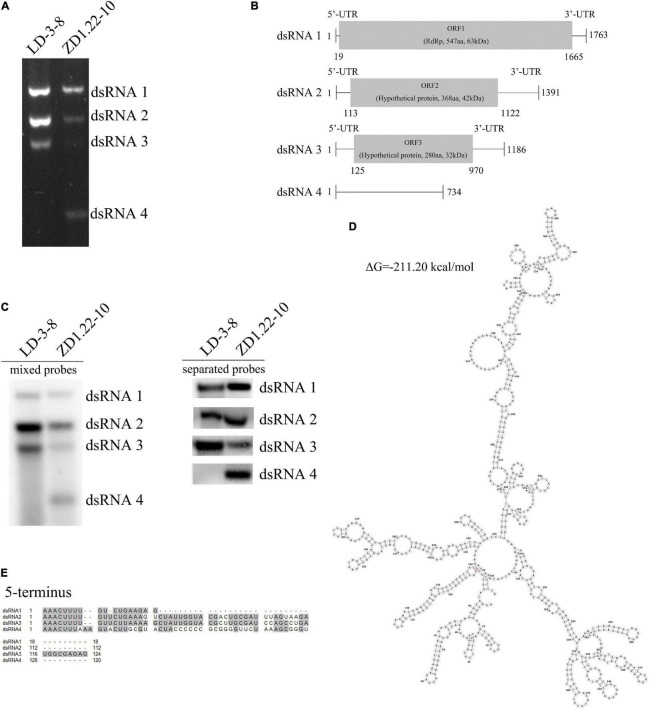
Genomic organization of AfPV1 and SatRNA. **(A)** Agarose gel electrophoresis of the dsRNA segments extracted from *A. flavus* isolate LD-3-8 and ZD1.22-10. **(B)** Genome organization of dsRNA1, dsRNA2, and dsRNA3 indicated by the diagrammatic representation. **(C)** Detection of dsRNA1, dsRNA2, dsRNA3, and dsRNA4 by northern blot. Probe-dsRNA1, probe-dsRNA2, probe-dsRNA3, and probe-dsRNA4 were mixed or used separately to detect RNAs. **(D)** Secondary structure analysis of the dsRNA4 using RNAstructure 6.3 (Lu et al., 2006) showed that ribonucleotides are involved in the formation of secondary structures and predicted the presence of stem-loop structures. **(E)** Identity at the 5′-terminal of dsRNA1, dsRNA2, dsRNA3, and dsRNA4. Gray shading indicates that nucleotides are identical in the three segments.

There were four dsRNA segments (dsRNA1, dsRNA, dsRNA3, and dsRNA4) detected in *A. flavus* isolate ZD1.22-10-9 (Figures 1A,B). We obtained nearly complete sequences of dsRNA1, dsRNA2, and dsRNA3 (GenBank sequences ON409581, ON409582, and ON409583 respectively). These three sequences shared more than 99% sequence identity with mycovirus AfPV1 (GenBank sequences MK344768, MK344769, and MK344770 respectively) (Jiang et al., 2019). We suggest that dsRNA1, dsRNA2 and dsRNA3 from *A. flavus* isolate ZD1.22-10-9 comprise the mycovirus AfPV1. The complete sequences of dsRNA4 (66.7% GC) did not contain any ORFs, indicating a lack of encoded proteins (Figure 1B). It has been deposited in GenBank with accession OM650825. A sequence similarity search (BLASTn) of the GenBank database revealed no significant homology between dsRNA4 and other sequences. Sequence analyses showed conserved nucleotides (AAACUUU) at the 5′-terminus of dsRNA1, dsRNA2, dsRNA3, and dsRNA4 (Figure 1E), suggesting that dsRNA4 may be using the RNA-dependent RNA polymerase (RdRp) encoded by AfPV1 to replicate (Nibert et al., 2014). There was little or no sequence conservation at the 3′-termini (Supplementary Figure S1). Secondary structure analysis of dsRNA4 revealed that 65% of the nucleotides were involved in the formation of stem-loop structures (Figure 1D), which are commonly found in mycoviruses and related to RdRp recognition and replication (Ghabrial and Suzuki, 2009).

**FIGURE 2:**
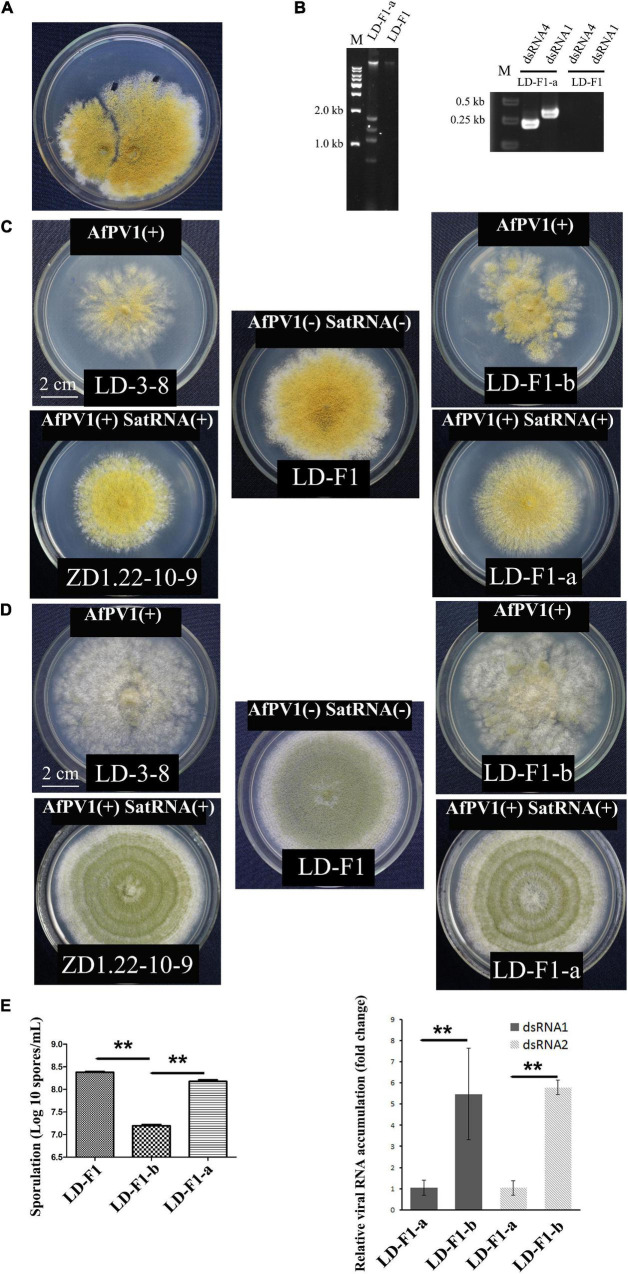
Effects of AfPV1 and satellite RNA in *A. flavus.* **(A)** Paired-cultures between the donor isolate ZD1.22-10-9 (left) and the virus-free recipient isolate LD-F1 (right). Derivative isolates were obtained from the mycelial agar plugs of LD-F1. **(B)** Agarose gel electrophoresis of dsRNA extracted from derivative isolates (left), and RT-PCR detection for AfPV1 and satellite RNA (right). Colony morphology of isolates LD-3-8, ZD1.22-10-9, LD-F1, LD-F1-a, and LD-F1-b after culturing on CZ **(C)** and PDA **(D)** for 6 days. **(E)** Sporulation of isolates LD-F1, LD-F1-b, and LD-F1-a (left). The accumulation of AfPV1 in isolates LD-F1-a and LD-F1-b (right). Isolate LD-3-8 was infected with AfPV1, virus-free isolate LD-F1 was obtained from isolate LD-3-8 by single sporing, and then was labeled with a pyrithiamine resistance (*ptr*) gene, and isolate LD-F1-b was one of the derivative isolates, obtained by transferring AfPV1 from isolate LD-3-8 (donor) to the virus-free isolate LD-F1 (recipient). Isolate ZD1.22-10-9 was infected with AfPV1 and satellite RNA, while isolate LD-F1-a was one of the derivative isolates, obtained by transferring AfPV1 and satellite RNA from isolate ZD1.22-10-9 (donor) to the virus-free isolate LD-F1 (recipient). *P < 0.05, **P < 0.01, ***P < 0.001, by Tukey’s multiple comparison tests.

The bands were assessed by treatments with DNase I, RNase A, or S1 nuclease. The dsRNA1, dsRNA2, dsRNA3, and dsRNA4 were completely digested by RNase A, but not digested by DNase I or S1 nuclease (Supplementary Figure S2). These results strongly support that these were indeed dsRNAs.

### dsRNA4 Is the SatRNA of Helper Virus AfPV1 Infecting *Aspergillus flavus*

Northern blotting also shows that AfPV1 (dsRNA1, dsRNA2 and dsRNA3) was present in isolates LD-3-8 and ZD1.22-10-9 (Figure 1C). dsRNA4 was found in ZD1.22-10-9, but not in isolate LD-3-8 (Figure 1C). We tried to separate dsRNA4 from AfPV1 using dsRNA horizontal transmission and asexual single-spore isolation techniques. From these attempts, 78 single-spore isolates of ZD1.22-10-9 were obtained, and in all single-spore isolates, the segments dsRNA1, dsRNA2, dsRNA3, and dsRNA4 were detected. Attempts at horizontal transmission were made five times, and the results showed that dsRNA4 was found together with dsRNA1, dsRNA2 and dsRNA3 (AfPV1) in single isolates three times, and dsRNA1, dsRNA2 and dsRNA3 together were detected in a single isolate once. Single dsRNAs were not detected separately. Furthermore, we extracted and purified viral particles from isolate ZD1.22-10-9, and found that the viral particles contained dsRNA1, dsRNA2, dsRNA3, and dsRNA4, and these corresponded to dsRNA elements from mycelium of isolate ZD1.22-10-9 (Supplementary Figure S3). dsRNA4 was always detected with AfPV1, but AfPV1 could be isolated without dsRNA4. Therefore, dsRNA4 is a SatRNA of helper virus AfPV1 infecting *A. flavus*.

### Effects of AfPV1 and SatRNA on Colony Morphology and Sporulation of *Aspergillus flavus*

**FIGURE 3:**
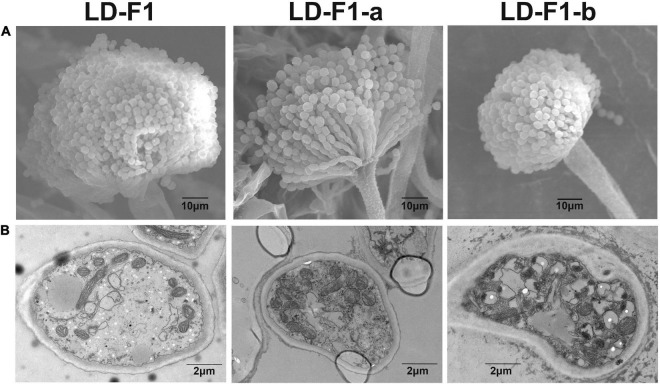
**(A)** Conidial head of virus-free isolate (LD-F1), AfPV1-infected isolate (LD-F1-b), and AfPV1- and SatRNA-infected isolate (LD-F1-a) were observed by scanning electron microscopy (SEM). **(B)** Morphology of vacuoles in virus-free isolate (LD-F1), AfPV1-infected isolate (LD-F1-b), and AfPV1- and SatRNA-infected isolate (LD-F1-a) was observed by transmission electron microscope (TEM).

Isolate LD-F1-a infected with AfPV1 and SatRNA (dsRNA4), was obtained from a recipient colony of LD-F1 (Figure 2A). The infection with AfPV1 and SatRNA (dsRNA4) was confirmed by dsRNA extraction and RT-PCR amplification (Figure 2B). Compared to the original virus-free isolate LD-F1 (Jiang et al., 2019), AfPV1 infection caused abnormal colony morphology and poor sporulation (Figures 2C–E). But SatRNA (dsRNA4) infection could allow recovery from the symptoms caused by AfPV1 (Figures 2C–E). Moreover, SatRNA (dsRNA4) could also significantly reduce the accumulation of AfPV1 in *A. flavus* (Figure 2E). The conidial heads of isolate LD-F1 (virus-free) and isolate LD-F1-a (AfPV1- and SatRNA-infected) were larger than those of isolate LD-F1-b (AfPV1-infected) (Figure 3A), suggesting poor sporulation of isolate LD-F1-b. We also noticed that the size of vacuoles in LD-F1-b (AfPV1-infected) was larger and more than that in the LD-F1 (virus-free) and isolate LD-F1-a (AfPV1- and SatRNA-infected), suggesting that AfPV1 influences the size and number of vacuoles in *A. flavus* (Figure 3B).

### Effects of AfPV1 and SatRNA on Stress Tolerance of *Aspergillus flavus*

**FIGURE 4:**
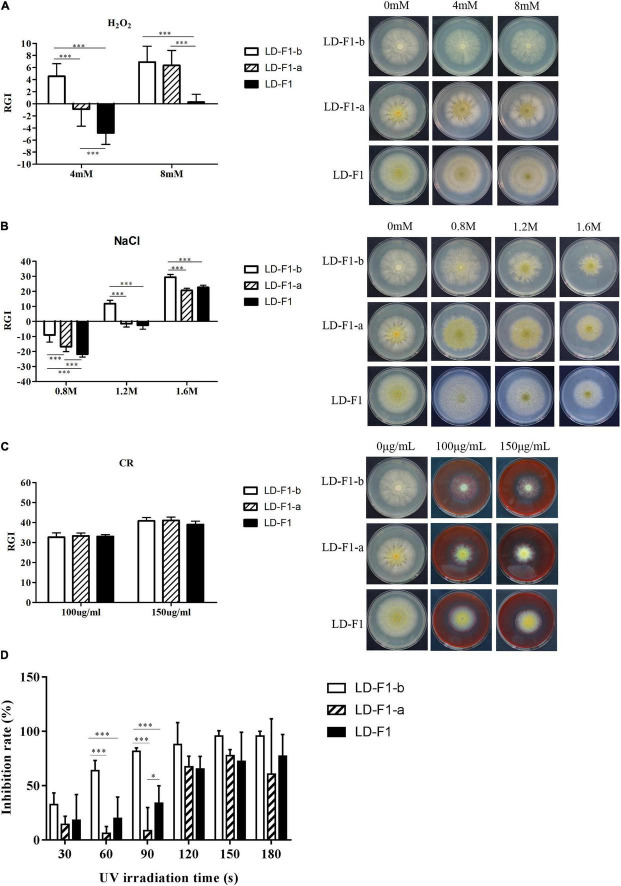
Comparisons of tolerance to stresses by virus-free isolate (LD-F1), AfPV1-infected isolate (LD-F1-b), and AfPV1- and SatRNA-infected isolate (LD-F1-a). Radial growth inhibition under oxidative stress **(A)**, osmotic stress **(B)**, cell wall stress **(C)**, UV stress **(D)**. *P < 0.05, **P < 0.01, ***P < 0.001, by Tukey’s multiple comparison tests.

In order to observe effects on stress tolerance by AfPV1 and SatRNA in *A. flavus*, we examined the effects of several stresses on AfPV1-infected (LD-F1-b), AfPV1- and SatRNA-infected (LD-F1-a), and virus-free (LD-F1) lines. We found that infection by AfPV1 caused greater sensitivity to oxidative stresses in *A. flavus*, and SatRNA (dsRNA4) could restore some resistance under low oxidative stress conditions (4 mM H_2_O_2_), but could not restore the stress resistance under high oxidative stress (8 mM H_2_O_2_) (Figure 4A). Furthermore, infection by AfPV1 caused greater sensitivity to osmotic and UV stresses in *A. flavus*, and satRNA (dsRNA4) could ameliorate the loss of stress resistance (Figures 4B,D). However, there were no significant differences in cell wall stress (CR) resistance among AfPV1-infected, AfPV1- and SatRNA-infected, and virus-free isolates (Figure 4C). Moreover, we noticed that the AfPV1-infected isolate was extremely sensitive to UV irradiation, and when UV irradiation time reach 150 s, the conidia of the AfPV1-infected isolate showed 100% mortality (Figure 4D). But there was only a significant difference between AfPV1- and SatRNA-infected (LD-F1-a) and virus-free (LD-F1), when UV irradiation time was at 90 s (Figure 4D).

### Effect of AfPV1 and SatRNA on Pathogenicity of *Aspergillus flavus*

**FIGURE 5:**
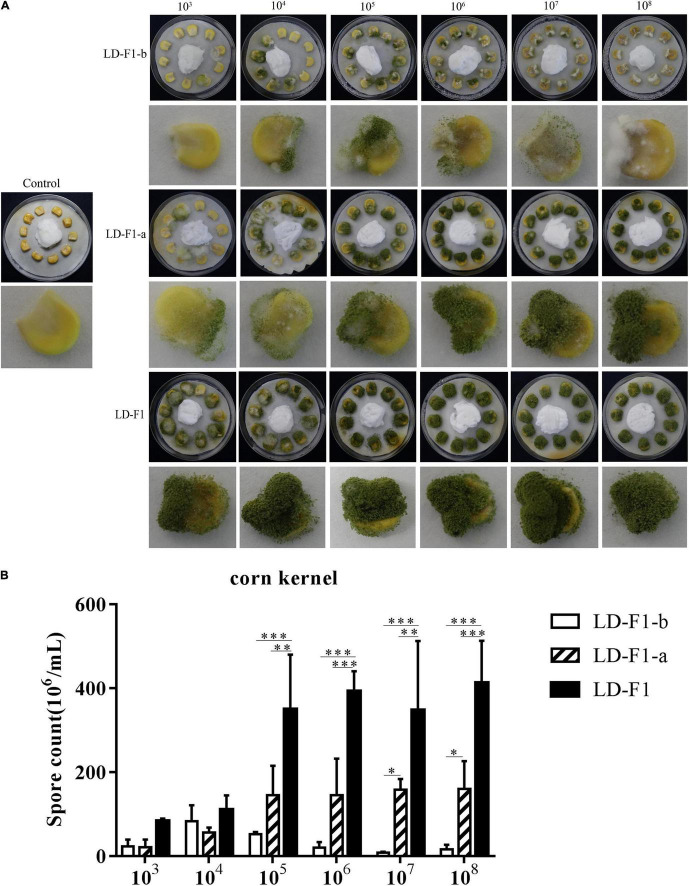
**(A)** Colonization of virus-free isolate (LD-F1), AfPV1-infected isolate (LD-F1-b), and AfPV1- and SatRNA-infected isolate (LD-F1-a) on maize kernel. **(B)** Conidia production of virus-free isolate (LD-F1), AfPV1-infected isolate (LD-F1-b), and AfPV1- and SatRNA-infected isolate (LD-F1-a) was assessed on infected maize kernels. *P < 0.05, **P < 0.01, ***P < 0.001, by Tukey’s multiple comparison tests.

In order to observe effects on pathogenicity by AfPV1 and SatRNA in *A. flavus*, we used several models to compare AfPV1-infected (LD-F1-b), AfPV1- and SatRNA-infected (LD-F1-a), and virus-free (LD-F1) isogenic lines. In the corn model, isolates LD-F1-b and LD-F1-a appeared to grow less vigorously than LD-F1, whereas LD-F1-b showed significantly the lowest vigor (Figure 5B). Sporulation was also measured for these isolates on corn seeds, and isolate LD-F1-a sporulated less that wild-type while LD-F1-b showed the least sporulation (Figure 5A).

**FIGURE 6:**
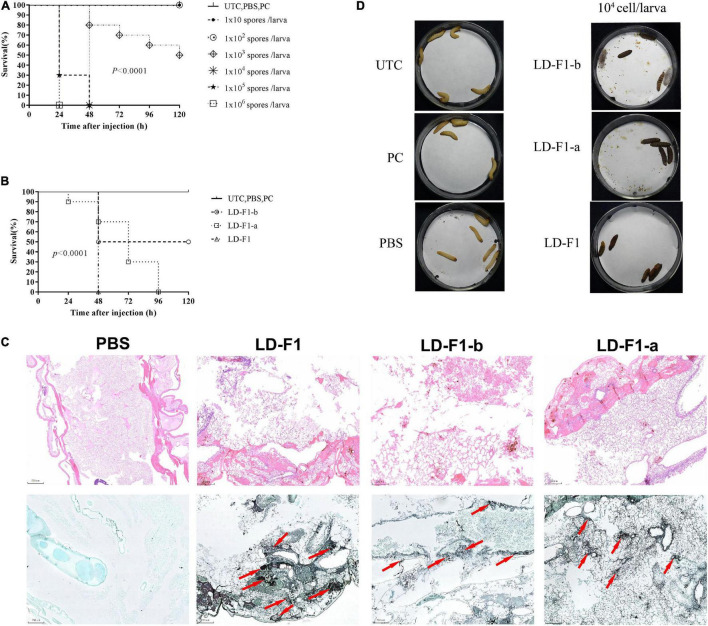
**(A)** Determination of the optimal spore concentration of *A. flavus* for pathogenicity testing in *G. mellonella.* The *G. mellonella* larvae were injected with spores of *A. flavus* isolate LD-F1 using 10, 10^2^, 10^3^, 10^4^, 10^5^, and 10^6^ spores per larva. **(B)** Survival of *G. mellonella* larvae infected with 10^5^ spores/larva of virus-free isolate (LD-F1), AfPV1-infected isolate (LD-F1-b), and AfPV1- and SatRNA-infected isolate (LD-F1-a) over a 120 h incubation period. **(C)** Histological observation at 48 h post-inoculation among tissues infected with the virus-free isolate (LD-F1), AfPV1-infected isolate (LD-F1-b), and AfPV1- and SatRNA-infected isolate (LD-F1-a), and the HE staining is on top, while the GMS staining is below, and the red arrows indicate hyphae growth. **(D)** Melanization of larvae infected with the virus-free isolate (LD-F1), AfPV1-infected isolate (LD-F1-b), and AfPV1- and SatRNA-infected isolate (LD-F1-a). Control experiments are comprised of non-treated larvae (UTC), pierced larvae (PC), and PBS-injected larvae (PBS). *P*-values were estimated using Log rank tests.

In order to determine the optimal spore concentration of *A. flavus* for pathogenicity tests, larvae of *G. mellonella* were injected with spores of LD-F1 from 10, 10^2^, 10^3^, 10^4^, 10^5^, to 10^6^ spores/larva. The infectious dose of 10^6^ spores/larva resulted in 0% survival within 24 h, while doses 10^2^ spores/larva or less were not infectious (Figure 6A). The dose of 10^3^ spores/larva achieved 50% mortality at 120 h (Figure 6A), and 10^4^ and 10^5^ could reach 100% mortality by 48 h (Figure 6A). Doses of 10^4^ and 10^5^ spores/larva were optimal to observe some possible differences in pathogenicity of *A. flavus* isolates, and 10^4^ spores/larva was chosen for pathogenicity tests. Melanization of larvae, which is an indicator of fungal growth and an active immune response, was observed in all inoculation treatments except for controls (UTC, PBS, and PC) (Figure 6D). Isolate LD-F1 at 10^4^ spores/larva could cause 100% mortality within 48 h. LD-F1-a at 10^4^ spores/larva could cause 100% mortality within 96 h, but LD-F1-b at 10^4^ spores/larva caused only 50% mortality within 120 h (Figures 6B,C). There were significant differences among the survival rates of larva infected with LD-F1, LD-F1-a, and LD-F1-b (*P* < 0.0001) (Figures 6B,C). Histological analyses at 48 h post-inoculation showed apparent differences among tissues infected with the virus-free isolate (LD-F1), AfPV1-infected isolate (LD-F1-b), and AfPV1- and SatRNA-infected isolate (LD-F1-a) (Figure 6C). The HE staining (Hematoxylin and eosin stain) of larvae revealed that those infected with the virus-free isolate (LD-F1) seemed to show more collapsed cells than those infected with the AfPV1-free isolate (LD-F1), or the AfPV1- and SatRNA-infected isolate (LD-F1-a), but there was no damage observed in the control (PBS) (Figure 6C). The GMS staining of the larvae revealed more hyphae in the larvae infected with the virus-free isolate (LD-F1) than the AfPV1- and SatRNA-infected isolate (LD-F1-a), much less hyphae in the larvae infected with the AfPV1-free isolate (LD-F1-b), and no hyphal growth was observed in the control (PBS) (Figure 6C).

**FIGURE 7:**
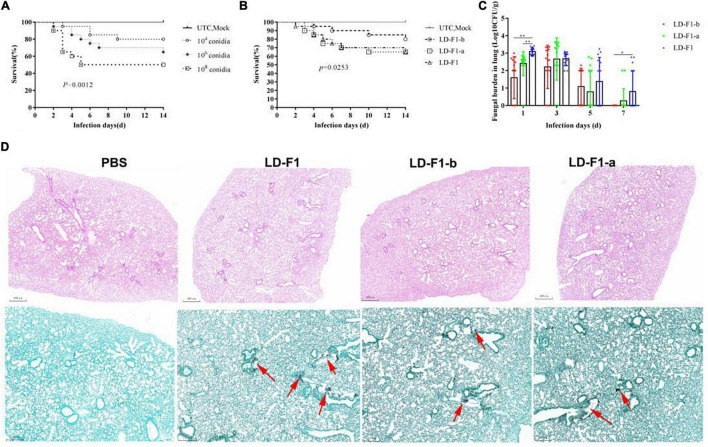
Comparison of virulence in mice of virus-free isolate (LD-F1), AfPV1-infected isolate (LD-F1-b), and AfPV1- and SatRNA-infected isolate (LD-F1-a) by intratracheal injection. **(A)** Determination of the optimal spore concentration of *A. flavus* for pathogenicity testing in immunosuppressed mice inoculated with 50 μL spores of *A. flavus* isolate LD-F1 ranging in concentration from 10^4^, 10^6^ to 10^8^ CFU/mL. **(B)** Survival of mice inoculated with spores of virus-free isolate (LD-F1), AfPV1-infected isolate (LD-F1-b), and AfPV1- and SatRNA-infected isolate (LD-F1-a) over a 14 days incubation period. **(C)** Fungal burden in lung tissue over 7 days. **(D)** Histological observation at 3 days post-inoculation in lung tissue infected with the virus-free isolate (LD-F1), AfPV1-infected isolate (LD-F1-b), and AfPV1- and SatRNA-infected isolate (LD-F1-a), and the HE staining is on top, while the GMS stain is below, and the red arrows indicate hyphae growth. Control experiments are comprised of untouched mice (UTC) and saline buffer injected immunosuppressive mice (Mock). *P*-values were estimated using Log rank, non-parametric Kruskal–Wallis and Dunn’s multiple comparison tests. *P < 0.05, **P < 0.01, ***P < 0.001.

In order to determine the optimal spore concentration of *A. flavus* for pathogenicity tests in mice, immunosuppressed ICR mice were inoculated with spores of *A. flavus* isolate LD-F1 in concentrations of 10^4^, 10^6^, and 10^8^ CFU/mL. In the intratracheal injection experimental group, each immunosuppressed mouse was anesthetized by injection of chloral hydrate and intravenously inoculated with 50 μL of each conidial suspension via intranasal instillation. The 10^4^ CFU/mL treatment caused 20% mortality within 14 days, and the 10^6^ and 10^8^ CFU/mL treatments caused 35% and 50% mortality, respectively (Figure 7A). The controls (UTC and Mock) did not cause any mortality (Figure 7A). With these results in mind, 10^6^ CFU/ml was chosen as the optimal dose since its intermediate pathogenicity level facilitated determination of differences in the virulence of different isolates. Compared with LD-F1 and LD-F1-a, the pathogenicity of LD-F1-b was significantly reduced in this infection model (Figure 7B), while there were no noticeable differences between LD-F1 and LD-F1-a (Figure 7B). We also measured pulmonary fungal burden on days 1, 3, 5, and 7 after inoculation, and the amount gradually decreased with days after inoculation (Figure 7C). Moreover, the pulmonary fungal burden at 1 and 7 days after inoculation was significantly lower in mice inoculated with the AfPV1-infected isolate (LD-F1-b) than in mice infected with the virus-free isolate (LD-F1), but no significant differences were found at other times (Figure 7C). Histological analyses at 72 h post-inoculation revealed no differences among lung tissues infected with the virus-free isolate (LD-F1), the AfPV1-infected isolate (LD-F1-b), or the AfPV1- and SatRNA-infected isolate (LD-F1-a) (Figure 7D). Hyphae were observed in the lung tissues inoculated with any isolate of *A. flavus*, but no hyphal growth was observed in the mock control (Figure 7D).

**FIGURE 8:**
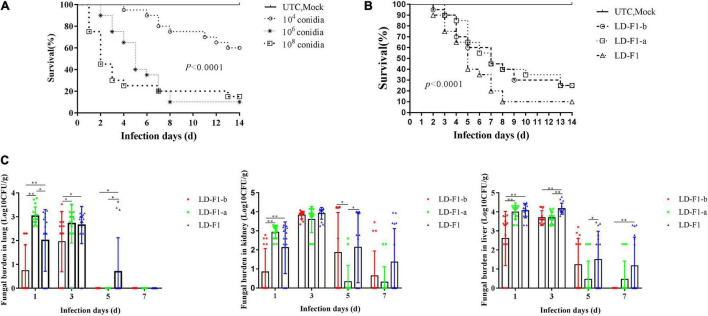
Comparisons of virulence in mice of virus-free isolate (LD-F1), AfPV1-infected isolate (LD-F1-b), and AfPV1- and SatRNA-infected isolate (LD-F1-a) by lateral tail vein injection. **(A)** Determination of the optimal spore concentration of *A. flavus* for pathogenicity testing in mice and the immunosuppressed mice were infected with 40 μL spores of *A. flavus* isolate LD-F1 ranging in concentration from 10^4^, 10^6^ to 10^8^ CFU/mL. **(B)** Survival of mice infected with spores of virus-free isolate (LD-F1), AfPV1-infected isolate (LD-F1-b), and AfPV1- and SatRNA-infected isolate (LD-F1-a) over a 14 days incubation period. **(C)** Fungal burden in lung, kidney, and liver tissue over 7 days. Control experiments are comprised of untouched mice (UTC) and saline buffer injected immunosuppressive mice (Mock). *P*-values were estimated using Log rank, non-parametric Kruskal–Wallis and Dunn’s multiple comparison tests. *P < 0.05, **P < 0.01, ***P < 0.001.

In the experiment on injection via lateral tail vein with 40 μl of each conidial suspension, 10^4^ CFU/mL caused only 40% mortality within 14 days, but 10^6^ CFU/mL and 10^8^ CFU/mL allowed 10% and 15% survival (no significant difference), respectively (Figure 8A), and the Control (UTC and Mock) did not cause any mortality (Figure 8A). So the dose of 10^6^ CFU/mL was chosen as the optimal spore concentration of *A. flavus* for pathogenicity tests. Compared with LD-F1, the mouse survival rate by LD-F1-b and LD-F1-a were significantly higher, but no significant differences were found between the latter two (Figure 8B). However, the fungal burden was significantly lower in mice inoculated with the AfPV1-infected isolate (LD-F1-b) than in mice infected with the AfPV1 and SatRNA-infected isolate (LD-F1-a) in lung, liver, and kidney tissues (Figure 8C), indicating that the pathogenicity of LD-F1-b was even weaker than LD-F1-a. Moreover, the tissues of lung, liver, and kidney were also taken for histological analyses at 72 h post-inoculation, and no differences among tissues inoculated with the virus-free isolate (LD-F1), AfPV1-infected isolate (LD-F1-b), or AfPV1- and SatRNA-infected isolate (LD-F1-a) were seen (Supplementary Figure S4).

### Adherence and Mortality Assay

**FIGURE 9:**
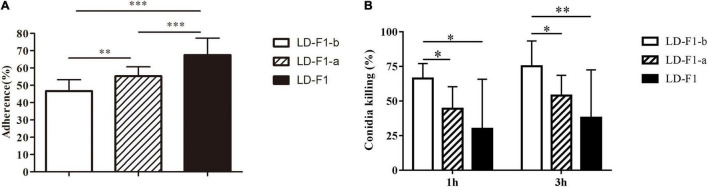
**(A)** Adherence of conidia of virus-free isolate (LD-F1), AfPV1-infected isolate (LD-F1-b), and AfPV1- and SatRNA-infected isolate (LD-F1-a) to A549 human pneumocyte cells. **(B)** Conidial killing of virus-free isolate (LD-F1), AfPV1-infected isolate (LD-F1-b), and AfPV1- and SatRNA-infected isolate (LD-F1-a) to at 1 and 3 h phagocytosis by RAW264.7, a murine macrophage cell line. *P*-values were estimated using Tukey’s multiple comparisons, non-parametric Kruskal–Wallis and Dunn’s multiple comparison tests. *P < 0.05, **P < 0.01, ***P < 0.001.

The conidial adherence of *A. flavus* to lung epithelial cells and conidial mortality of *A. flavus* cultured with macrophages were investigated. The AfPV1-infected isolate (LD-F1-b) reduced adherence of the conidia to host epithelial cells of the type II human pneumocyte cell line A549 in comparison with the adherence of the AfPV1- and SatRNA-infected isolate (LD-F1-a) and virus-free isolate (LD-F1) (Figure 9A). And AfPV1- and SatRNA-infected isolate (LD-F1-a) reduced adherence of the conidia to host epithelial cells of the type II human pneumocyte cell line A549 in comparison to the virus-free isolate (LD-F1) (Figure 9A). Moreover, conidia from the AfPV1-infected isolate (LD-F1-b) showed a reduced survival rate after co-culturing with macrophage RAW264.7 cells compared with the survival rate of the virus-free isolate (LD-F1) and the AfPV1- and SatRNA-infected isolate (LD-F1-a) (Figure 9B). Also, conidia from the AfPV1- and SatRNA-infected isolate (LD-F1-a) showed a reduced survival rate after co-culturing with macrophage RAW264.7 cells compared with the survival rate of the virus-free isolate (LD-F1) (Figure 9B).

### Differential Gene Expression of *Aspergillus flavus* in Response to Infection by AfPV1 and SatRNA

**FIGURE 10:**
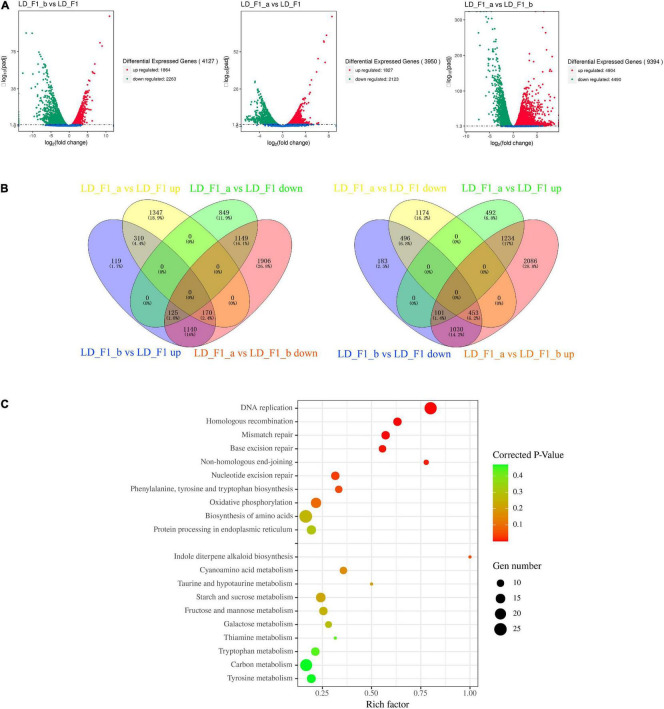
*Aspergillus flavus* genes that were differentially expressed in response to AfPV1 and Satellite RNA that were identified by RNA-Seq. **(A)** Volcano plot of RNA-Seq data using Log 2 fold change and Log 10 (padj). *X* and *Y* axes represent Log 2 -converted fold change and Log 10 -converted padj. **(B)** Venn diagrams illustrating the number of genes that were differentially expressed in subsets of the virus-free isolate (LD-F1), AfPV1-infected isolate (LD-F1-b), and AfPV1- and SatRNA-infected isolate (LD-F1-a). **(C)** Bubble chart for KEGG pathway enrichment analysis of different expression in response to AfPV1 and Satellite RNA.

Here, we investigated genome-wide transcriptional differences in *A. flavus* expression in response to the infections by either AfPV1 or AfPV1-satRNA (dsRNA4). Compared with the LD-F1 isolate, LD-F1-b up-regulated 1864 genes, and down-regulated 2263 genes (Figure 10A, LD-F1-b vs. LD-F1). Compared with LD-F1, LD-F1-a up-regulated 1827 genes, and down-regulated 2123 genes (Figure 10A, LD-F1-a vs. LD-F1). Compared with LD-F1-b, LD-F1-a up-regulated 4904 genes, and down-regulated 4490 genes (Figure 10A, LD-F1-a vs. LD-F1-b).

As the results showed, AfPV1-infection caused severe debilitation symptoms in *A. flavus*, and SatRNA (dsRNA4) could attenuate these symptoms. So, we searched and obtained the 1435 (125 + 1140 + 170) genes up-regulated by AfPV1 (LD-F1-b vs. LD-F1 up), but also down-regulated by SatRNA (LD-F1-a vs. LD-F1-b down) (Figure 10B). Furthermore, we found 125 genes that were up-regulated by AfPV1 (LD-F1-b vs. LD-F1 up) but over down-regulated by SatRNA (LD-F1-a vs. LD-F1 down), 170 genes that were up-regulated by AfPV1 (LD-F1-b vs. LD-F1 up) but slightly down-regulated by SatRNA (LD-F1-a vs. LD-F1 up), and 1140 genes that were up-regulated by AfPV1 (LD-F1-b vs. LD-F1 up) but down-regulated by SatRNA to the level of LD-F1 (virus-free) (Figure 10B). We searched and obtained the 1584 genes (101 + 1030 + 453) down-regulated by AfPV1 (LD-F1-b vs. LD-F1 down), but also up-regulated by SatRNA (LD-F1-a vs. LD-F1-b up) (Figure 10B). Among these, 101 were down-regulated by AfPV1 (LD-F1-b vs. LD-F1 down) but over up-regulated by SatRNA (LD-F1-a vs. LD-F1 up); 170 were down-regulated by AfPV1 (LD-F1-b vs. LD-F1 down) but slightly up-regulated by SatRNA (LD-F1-a vs. LD-F1 down); and 1140 were down-regulated by AfPV1 (LD-F1-b vs. LD-F1 up) but up-regulated by SatRNA to the level of LD-F1 (virus-free) (Figure 8B).

The 1435 genes (up-regulated by AfPV1, but also down-regulated by SatRNA), and the 1584 genes (down-regulated by AfPV1, but also up-regulated by SatRNA) were analyzed for the frequency of occurrence in each InterPro domain, and the 20 most abundant InterPro domains are shown in Table 1. The genes that were up-regulated by AfPV1 and down-regulated by SatRNA were mostly related to P-loop containing nucleoside triphosphate hydrolase (IPR027417), Alpha/beta hydrolase fold-1 (IPR000073), Helicase, C-terminal (IPR001650), including AAA + ATPase domain (IPR003593; AAA + ATPase domain; IPR003959: ATPase, AAA-type, core), and Pectin lyase fold (IPR011050: Pectin lyase fold/virulence factor; IPR012334: Pectin lyase fold). The genes that were down-regulated by AfPV1 and up-regulated by SatRNA were mostly related to Major facilitator superfamily (IPR020846: Major facilitator superfamily domain; IPR011701: Major facilitator superfamily), NAD(P)-binding domain (IPR016040: NAD(P)-binding domain; IPR023753: Pyridine nucleotide-disulphide oxidoreductase, FAD/NAD(P)-binding domain), and fungal transcription factor (IPR001138: Zn(2)-C6 fungal-type DNA-binding domain; IPR007219: Transcription factor, fungi).

**TABLE 1:** The most abundant InterPro domains according to the InterProScan.

InterPro ID	The genes that up-regulated by AfPV1 and down-regulated by SatRNA	Count	InterPro ID	The genes that down-regulated by AfPV1 and up-regulated by SatRNA	Count
IPR027417	P-loop containing nucleoside triphosphate hydrolase	63	IPR020846	Major facilitator superfamily domain	151
IPR003593	AAA + ATPase domain	23	IPR011701	Major facilitator superfamily	111
IPR013785	Aldolase-type TIM barrel	18	IPR016040	NAD(P)-binding domain	104
IPR000073	Alpha/beta hydrolase fold-1	15	IPR023753	Pyridine nucleotide-disulfide oxidoreductase, FAD/NAD(P)-binding domain	52
IPR001650	Helicase, C-terminal	13	IPR001138	Zn(2)-C6 fungal-type DNA-binding domain	50
IPR011050	Pectin lyase fold/virulence factor	13	IPR001128	Cytochrome P450	45
IPR012334	Pectin lyase fold	13	IPR007219	Transcription factor, fungi	42
IPR003959	ATPase, AAA-type, core	12	IPR002347	Glucose/ribitol dehydrogenase	31
IPR005123	Oxoglutarate/iron-dependent dioxygenase	11	IPR017972	Cytochrome P450, conserved site	29
IPR012340	Nucleic acid-binding, OB-fold	10	IPR005828	General substrate transporter	29
IPR001810	F-box domain, cyclin-like	9	IPR002401	Cytochrome P450, E-class, group I	25
IPR001199	Cytochrome b5-like heme/steroid binding domain	8	IPR011032	GroES-like	25
IPR000330	SNF2-related	8	IPR002085	Alcohol dehydrogenase superfamily, zinc-type	23
IPR001926	Tryptophan synthase beta subunit-like PLP-dependent enzymes superfamily	8	IPR003663	Sugar/inositol transporter	22
IPR013057	Amino acid transporter, transmembrane	7	IPR005829	Sugar transporter, conserved site	21
IPR001357	BRCT domain	7	IPR013154	Alcohol dehydrogenase GroES-like	20
IPR018506	Cytochrome b5, heme-binding site	6	IPR002293	Amino acid/polyamine transporter I	20
IPR001461	Peptidase A1	5	IPR013149	Alcohol dehydrogenase, C-terminal	20
IPR008921	DNA polymerase III, clamp loader complex, gamma/delta/delta subunit, C-terminal	5	IPR013785	Aldolase-type TIM barrel	20
IPR005103	Glycoside hydrolase, family 61	5	IPR020843	Polyketide synthase, enoylreductase	17
IPR021109	Aspartic peptidase	5	IPR002938	Monooxygenase, FAD-binding	16
IPR000262	FMN-dependent dehydrogenase	5	IPR020904	Short-chain dehydrogenase/reductase, conserved site	16
IPR012133	Alpha-hydroxy acid dehydrogenase, FMN-dependent	5	IPR017871	ABC transporter, conserved site	13

Furthermore, these genes were also submitted to the KOBAS database^3^ for KEGG pathway analysis. Interestingly the 1435 genes (up-regulated by AfPV1, but also down-regulated by SatRNA) were significantly enriched in these functions: Non-homologous end-joining, Homologous recombination, DNA replication, Mismatch repair, Base excision repair, and Nucleotide excision repair (Figure 10C, Corrected *P*-value < 0.1), and these pathways were mostly related to genomic DNA stability of eukaryotes (Wyman and Kanaar, 2006; Michel et al., 2007). The 1584 genes (down-regulated by AfPV1, but also up-regulated by SatRNA) were significantly enriched in the pathway of Indole diterpene alkaloid biosynthesis (Figure 10C, Corrected *P*-value < 0.1). Some genes in these pathways (Non-homologous end-joining, Homologous recombination, DNA replication, Mismatch repair, Base excision repair, Nucleotide excision repair, and Indole diterpene alkaloid biosynthesis) were randomly selected and their expression in qRT-PCR was measured (Supplementary Figure S5). Their results were in agreement with transcriptional analysis.

## Discussion

*Aspergillus flavus* is an important fungal pathogen, which has been frequently reported as the second major cause of invasive aspergillosis (IA) in immunosuppressed patients (Rudramurthy et al., 2019). Invasive aspergillosis has been rising with the increasing number of immunosuppressed patients in the past few decades, especially in certain tropical countries (Brown et al., 2012). Unfortunately, only three categories of antifungal drugs are used to treat IA, such as polyenes (amphotericin B), triazoles (itraconazole, voriconazole and posaconazole), and echinocandins (caspofungin and micafungin) (Perlin et al., 2017). Moreover, antifungal antibiotics have some toxicity to humans, and control of *A. flavus* becomes more difficult with antibiotic resistance to current antifungal drugs (Paul et al., 2015; Espinel-Ingroff et al., 2021; Yang et al., 2021). Mycoviruses selectively infect fungi, and have the potential for treatment of pathogenic fungal infections (Van De Sande et al., 2010; Bhatti et al., 2011; Özkan and Coutts, 2015). In our research, we tested the use of mycoviruses as therapeutic strategies for *A. flavus* infection. We isolated several mycoviruses infecting *A. flavus*, and one of them (AfPV1) is a new member of *Partitiviridae* family (Jiang et al., 2019). *A. flavus* is a fungal pathogen of animals and plants, and we tested corn, honeycomb moths, and mice as models to examine the pathogenicity of AfPV1 to *A. flavus*. AfPV1 caused hypovirulence in *A. flavus*, which revealed the potential for therapy of *A. flavus* infections. Moreover, AfPV1 caused more sensitivity to osmotic, oxidative and UV stresses in *A. flavus*, and these stresses have also been found to be related to virulence factors (Amaike and Keller, 2011). Additionally, AfPV1 reduced the adhesion of conidia to host epithelial cells and increased conidial mortality by macrophages (Pasqualotto, 2009). The presence of the AfPV1 altered the colony morphology of *A. flavus*, and also lessened conidiophore numbers, resulting in decreased conidial numbers (Figures 2, 3A). Furthermore, TEM showed the presence of significantly larger vacuoles in AfPV1-infected cells (Figure 3B). We speculate that the aberrant vacuoles gave rise to the abnormal colony morphology in isolate LD-F1-b (AfPV1-infected), and dramatically altered *A. flavus* physiology. Animals which suffer from aspergillosis diseases of the lung, kidney, liver, spleen and brain are most often infected by inoculum as spores (Ford and Friedman, 1967; Smith, 1972). So, we confirmed the presence of *A. flavus* in the lungs, kidneys, and livers tissue of inoculated mice, which demonstrated the validity of the mouse infection assay. AfPV1-infected isolate ZD1.22-10-9 shares more than 99% sequence identity with mycovirus AfPV1-infected isolate LD-3-8, but we did not compared their effects on their hosts. *Cryphonectria hypovirus 1* (CHV1) with its genetic variability and diversity in its populations (nucleotide differences between subtypes ranged from 11 to 19%) (Gobbin et al., 2003). Krstin et al. (2020) found differences between CHV1 variants and growth rates of infected *C. parasitica* varying from 1.27 to 3.5 mm/day (Krstin et al., 2020).

This report describes the first discovery of a satellite dsRNA attenuating the helper virus-mediated symptoms in fungi. Satellite segments are categorized as either satellite viruses or satellite nucleic acids according to the capability of encoded structural proteins (Krupovic et al., 2016). In our study, dsRNA4 did not encode any protein, and may have been encapsidated instead by AfPV1-encoded capsid. In this situation, the dsRNA4 should be considered a satellite RNA of helper virus AfPV1. Conserved nucleotides (AAACUUU) were found at the 5′-termini of AfPV1 and dsRNA4, and a similar motif (AAACUUUUG/AU/G) is also present in the genomes of Botryosphaeria dothidea virus 1 (Jiang et al., 2019). Such conserved sequences of partitiviruses are often thought to be involved in RdRp recognition for RNA packaging and/or replication (Nibert et al., 2014). The secondary structure of dsRNA4 reveals that 65% of the ribonucleotides were involved in the formation of stem-loop structures (Figure 1D), which are commonly found in mycoviruses and related to RdRp recognition and replication (Ghabrial and Suzuki, 2009).

Members of *Partitiviridae* possess two essential genome segments: the larger one usually encodes RdRp, while the smaller one usually encodes the CP (Vainio et al., 2018). However, some additional dsRNA segments may also be present in viral genomes of *Partitiviridae* (Vainio et al., 2018). AfPV1 with three dsRNA segments, is a member of a new genus (“Zetapartitivirus”) in the *Partitiviridae* family (Jiang et al., 2019). dsRNA3 is one of the genomic segments of AfPV1 (Jiang et al., 2019), but dsRNA4 is not and can be separated from AfPV1. In our study, dsRNA4 was found to be a satellite RNA which relied on its helper virus AfPV1. A number of satellite RNAs dependent on various members of the *Partitiviridae* family have also been noted (Oh and Hillman, 1995; Kim et al., 2005). A satellite RNA with 677 bp in length, contained a small ORF and showed a conserved sequence motif (CGTAAAA) at the 5′-terminus with its helper virus Penicillium stoloniferum virus F (Kim et al., 2005), while dsRNA4 did not contain any ORFs and showed conserved nucleotides (AAACUUU) at the 5′-terminus with the helper virus AfPV1. Satellites can influence the accumulation and pathogenesis of their associated helper viruses. Helper viruses are responsible for maintenance of satellites in the host. For plant viruses, the functions of the satellite nucleic acids have been well studied, and they are able to modulate the symptoms caused by the helper viruses (Kaper and Waterworth, 1977; Waterworth et al., 1979; Wang and Smith, 2015). Tobacco necrosis satellite virus attenuates induced symptoms of the helper virus Tobacco necrosis virus, genus *Betanecrovirus*, family *Tombusviridae* (Gnanasekaran and Chakraborty, 2018). However, the biological functions of satellite nucleic acids are rarely reported for mycoviruses. For example, M1 is a satellite RNA of helper viruses in the *Totiviridae* family, which encodes the killer toxin lethal for *S. cerevisiae* cells that are free of M1 satellites (Gnanasekaran and Chakraborty, 2018). In our previous work, AfPV1 was found as a double-stranded RNA virus of the family *Partitiviridae* infecting *A. flavus* (Jiang et al., 2019). In this study, we found that AfPV1 infection caused abnormal colony morphology, poor sporulation, more sensitivity to some stresses, and reduced pathogenicity of *A. flavus.* The satellite RNA of helper virus AfPV1 was found as a double-stranded RNA (734 bp), which could attenuate the AfPV1-induced symptoms and reduce the accumulation of AfPV1 in *A. flavus*. The ability of satellite RNAs to reduce the helper virus-mediated symptoms has generally coincided with a reduction in the level of accumulation of the helper virus in infected host cells (Babos and Kassanis, 1962; Francki et al., 1986; Palukaitis, 2016). A similar satellite RNA (439 bp) has been found in *A. foetidus* (Shah et al., 2015), but its biological functions are not well studied.

Viruses with their limited coding capacity often harness host cellular factors to generate progeny virions (Luftig, 2014). Moreover, the smaller the viral genome, the less coding capacity, and the greater the need to use host cellular processes (Weitzman and Fradet-Turcotte, 2018). DNA viruses hijack and manipulate host DNA replication and DNA damage response processes to complete their life cycles in host cell, which can compromise host usage of the cellular machinery (Turnell and Grand, 2012; Weitzman and Fradet-Turcotte, 2018). In our research, the most enriched KEGG pathways of the genes up-regulated by AfPV1 were related to DNA replication and DNA damage response processes. This was in accordance with the observation that the AfPV1-infected isolate was extremely sensitive to UV irradiation (Figure 4D), and might indicate that AfPV1 utilized cellular processes to complete the replication in *A. flavus*. Activation and manipulation of the host DNA damage response processes by DNA viruses and single strand RNA viruses have been extensively studied (Ryan et al., 2016). However, dsRNA viruses that induce significant DNA damage have rarely been reported (Ryan et al., 2016). Retroviral RNA viruses reverse transcribe their RNA genome and integrate the subsequent DNA intermediate into the chromosomes of host cells, which can also activate the DNA damage response in host cell (Daniel et al., 1999). It is reported that the NHEJ pathway has a central role in post-integration DNA repair for host DNA damage responses to retroviral replication (Daniel et al., 1999, 2004; Li et al., 2001; Lau et al., 2004). We found that the key NHEJ genes (Ku70: CADAFLAP00006756 and Ku80: CADAFLAP00007225) were also up-regulated by AfPV1. However, Non-retroviral RNA virus sequences are widespread in the genomes of vertebrates, fungi, and plants (Chiba et al., 2011; Kryukov et al., 2019; Gilbert and Belliardo, 2021). Due to the lack of an integrase and an integration process, the horizontal transfer of genetic sequences from non-retroviral viruses to eukaryotic genomes is thought to occur accidentally during infection (Feschotte and Gilbert, 2012; Kryukov et al., 2019). In our study, we found that the pathways of the genes regulated by AfPV1 in its host are similar to retroviral viruses. And these pathways may be of benefit to viral integration or endogenization in the genomes of its hosts. Similar pathways were also found to be regulated by Sclerotinia sclerotiorum hypovirulence-associated DNA virus 1 (Qu et al., 2021). Moreover, the genes (down-regulated by AfPV1, but also up-regulated by SatRNA) were significantly enriched in pathway for Indole diterpene alkaloid biosynthesis (Figure 10C). A number of indole-diterpenes isolated from *Penicillium* spp. and *Aspergillus* spp. show significant activity against the H1N1 virus (Fan et al., 2013; Reddy et al., 2019; Zhou et al., 2021). Hence, we speculate that AfPV1 can ameliorate the production or the activity of antiviral substances from *A. flavus.*

In conclusion, we found a small double-stranded (ds) RNA segment (734 bp), which is a satellite RNA of its helper virus, AfPV1, infecting *A. flavus.* AfPV1-infection altered the colony morphology, decreased the number of conidiophores, created significantly larger vacuoles, and caused more sensitivity to osmotic, oxidative, and UV stresses in *A. flavus*. Moreover, AfPV1 infection reduced the pathogenicity of *A. flavus*, and also reduced the adhesion of conidia to host epithelial cells and increased conidial death by macrophages. However, the associated satellite RNA reduced the genomic accumulation of the helper virus AfPV1 in *A. flavus*, and also attenuated symptoms of the helper virus AfPV1 in *A. flavus.* Transcriptional analysis implied that the following pathways may be involved in regulation of the AfPV1 and the satellite RNA segment infection in *A. flavus*: Non-homologous end-joining, Homologous recombination, DNA replication, Mismatch repair, Base excision repair, Nucleotide excision repair, and Indole diterpene alkaloid biosynthesis. This is first functional description of a satellite RNA of a mycovirus.

## Data Availability Statement

The datasets presented in this study can be found in online repositories. The names of the repository/repositories and accession number(s) can be found below: NCBI BioProject – PRJNA816993.

## Ethics Statement

The animal study was reviewed and approved by the Animal Care Welfare Committee of Guizhou Medical University.

## Author Contributions

YJ designed and analyzed the data and wrote the manuscript. BY collected and analyzed the data. XL and XT analyzed the mice aspergillosis model. QW provided the type II human pneumocyte cell line A549. BW, QZ, WY, XQ, and YJ gave some advises for the work. TH revised the manuscript. All authors contributed to the article and approved the submitted version.

## Conflict of Interest

The authors declare that the research was conducted in the absence of any commercial or financial relationships that could be construed as a potential conflict of interest.

## Publisher’s Note

All claims expressed in this article are solely those of the authors and do not necessarily represent those of their affiliated organizations, or those of the publisher, the editors and the reviewers. Any product that may be evaluated in this article, or claim that may be made by its manufacturer, is not guaranteed or endorsed by the publisher.

## Footnotes

1
https://www.ncbi.nlm.nih.gov/genome/360?genome_assembly_id=968150

2
https://david.ncifcrf.gov/tools.jsp

3
http://kobas.cbi.pku.edu.cn/kobas3/?t=1

## Supplementary Material

Supplementary Figure S1 Identity at the 3′-terminal of dsRNA1, dsRNA2, dsRNA3, and dsRNA4. Gray shading indicates where nucleotides are identical in the three segments.

Supplementary Figure S2 dsRNA1, dsRNA2, dsRNA3, and dsRNA4 bands were assessed by treatments with DNase?, RNase A, or S1 nuclease.

Supplementary Figure S3 Agarose gel electrophoresed analysis of dsRNA profiles from mycelia and viral particles.

Supplementary Figure S4 Histological observation at 72 h post-inoculation in lung, liver, and kidney tissue infected with the virus-free isolate (LD-F1), AfPV1-infected isolate (LD-F1-b), and AfPV1- and SatRNA-infected isolate (LD-F1-a); HE staining **(A)** and GMS staining **(B)**. Control experiments are comprised of non-treated mice (UTC) and saline buffer injected immunosuppressive mice (Mock).

Supplementary Figure S5 The genes in these pathways (Non-homologous end-joining, Homologous recombination, DNA replication, Mismatch repair, Base excision repair, Nucleotide excision repair, and Indole diterpene alkaloid biosynthesis of *A. flavus*) were randomly selected and expression confirmed by qRT-PCR.
