# Supplementary material for: A Satellite dsRNA Attenuates the Induction of Helper Virus-Mediated Symptoms in Aspergillus flavus
Source: Front Microbiol. 2022 May 31;13:895844. doi: 10.3389/fmicb.2022.895844 (PMC9195127; doi:10.3389/fmicb.2022.895844)
Supplement: Supplementary file 1 [file Table_1.DOCX]

Table S1

| Probe name | Sequence from 5' to 3' |
| --- | --- |
| Probe-dsRNA1 | 1 CCAGAAAAGA GCGTGGTTGC TGGAACTCCA GCAGACATGC ATATATCTGG GACAACTTGG  61 GAAGCTCTTC GTCCAACACG AACCACCCAG GATTGGCTCA AGATGGCATT GTATCCATCA  121 ACGTATGTAA GAGACGCAAA CATGTCGTTT CAACGCCTTC TCGGCATTGG AATAGCAGGC  181 GGCTTTTACG ATGCAAAGTA CTGCACTTTC TTTGAGTACT TTCAAACTGG ATACGATTGT  241 CAACATGGTC CGAACCTTCT TTCGTGGAAG CGATTACGAT GGTTAGAACC TGTCTTCGGC  301 CTGAACGACC TCCCGAAGAT TTACAAACAG AAGCGAGCTG TTACCAAGAT ACGTTCGCTT  361 CTCTGGGCTC CTTAGCCCAC GAGGTACACT GAAAGCCAGT AGCTACATTC TCTCGTAGCG  421 ACCGT |
| Probe-dsRNA2 | 1 AGGTGACTGA AGGACTCCGA AATGTTGTTG AAGAATTGTT CATCAACATG GCTATTGTCC  61 AGATGCCACG TTATGAAAAG GGCTCGTTGG CTCAACTTTC CGAAACCACC GATGACCTTA  121 CCTTCTCCCA GTTTCCTCTT TCACTGGCTG ACCTTACCGT CAGTGCAGCG TTCAAAGTGG  181 GTAGAAATCT GCGCCGCTTC CTTCGCGCAT CCCCAGAACA GGTTGGTTCT TCATTGCGAA  241 GTGACCTAAT CCGAAAATCT GTTCGTCGTC GAGCGTAATC TCTGATTATG CACCTTTTAA  301 CAACAGCTTC ACGACAAGTC TTTAAGCTAT GACAAATGGT TAAAGTTGCT ACGAGTTGTT  361 ATGTAACGAA AGGTCGGACG TAGTGCTATT CGTACGCACT ACTGGCTCGC CGGTCGAGGA  421 CAAAACGTTA C |
| Probe-dsRNA3 | 1 GTGTCTACGA CACGACCGAA TGGCTATGTC CGATGCCTGA ATGCGGGTTA TGCGCATTCA  61 TCCTAACCTT TACGAAGTTT ACGCCTGACG ATCTTCGTAA GCATCCCCCC AGAGATGGAT  121 GCCTTTTGGA GCGCCCACCA GAGTATCCTC TAGGCTCTAT TTTCTCATCT CTGCTATCAC  181 GCCCTGGCAG GATCAAACAC CCAGGACAAG CTTTTTGTAG GATAGCCGAA GCAACTGTCT  241 CGAACAGATC CCTTTCTCAT CGCATGTTTG CTTATCAACA TGGTGATGCT GATGACGACT  301 CACCGTCTGA TTCATCATCG CTTGGGTAAC TGCGACTTAG GTTAGGAGTC TTGTTCTAAC  361 ATAAAATTTA CAAAAACACA AAAACATATA AAAACTTATA AAACACCTAG ATCATTTCGT  421 GATTTAGTAC AAAACATTTG ATTTTTGAAC CCAGTACGCC GAAAGACGGA ACTGTGAGTG  481 TTGTGCTCAC AGGGACT |
| Probe-dsRNA4 | 1 GTCGATAATG AGGGGCGACA GTATTGGGAC ATTGGGAACT ACAACGTTTG TTCAAGCGGT  61 TAACTACCGT GTGTGAAGGC TGCGGCATGT AACATGGACT TGTTTGTAGG ATCTTTGTCT  121 TTCGGTCATG GAGGCCGACC CAACGACGGA CATAGCGCGA CGGCGGATCA AGGCTGACCC  181 TAGTAGTCAG ATGGAAACGA AGCAAGTAGC GACAGGTGCG ATAACATCAA TCTGAACCCG  241 TCCAGAAGTA TGCTCCTCTT GCTGTAGTGG CAGGAGTTCC TAGAGTTGCG AAAGAAGGAT  301 TACCATAGCA CTCGTCCCCT TGTGGGTGTA GCATTTTCGT GTTGTTGTGC ATTCATCCCG  361 TTATGATAAG CGTAGACTAC CACTCGGTCA CTCATCTCCC GAAAGGGG |
