# Supplementary material for: A Satellite dsRNA Attenuates the Induction of Helper Virus-Mediated Symptoms in Aspergillus flavus
Source: Front Microbiol. 2022 May 31;13:895844. doi: 10.3389/fmicb.2022.895844 (PMC9195127; doi:10.3389/fmicb.2022.895844)
Supplement: Supplementary file 2 [file Table_2.DOCX]

>dsRNA1-ZD1.22-10-9

1 GTCTGAAGAG ATGGTAGTGC GTCTCACGTC CAGTAAGACA TCTGAACCCT TCACCGCCTT

61 TCCGTTTGAT TACCCTGGTA AGAGCGAAGT TGCTAAATCT TTGGAGCGTA ATCCATGGTT

121 AAAACCGTTT CGATACATGA CCCATGTTGG TCAGTATCCC TATAAACGAG GAGTGTTGCA

181 GAACACTCAG AAGTACGATC CATTTATAAA TGAAGCACTT CGACATTTCG ATAACGACCT

241 GCGTAAGTCA CTGAAAGGTT TCACAAGAAC TCCTGGCGAT GAGTTCAAGC TTAGAGAAGC

301 TCTCAACAAA TACGATGCCC CCACCCGTAC TTTCGAACAC GTCTTTCGCA ATGACCCCAA

361 ACTTGAAAGA TGTTACAGGC AAGCGTATTC TGAGGTTTTC GACGAGTTTG CTCTCAACCG

421 AAAGGTAGTT CCGAAGTTTC CTACCGCTGT TGACTTGGTT ATGGATTCAT CCTCTGGGTA

481 TCCGCATTTT CAAAAGAAAA GTGAAATTCG CGATCAAATA CTACACGAAG GACGAACCTG

541 GTTACACCAC GCCAAAAGCA AGGATTTTCA CCGAGTACCT CTGCTTCCAT GCAGTGTCGG

601 TGTACGTGGC GCTCTGTCAC CAGAGAACGA CCCAAAGACA AGACTCGTGT GGATGTACCC

661 TGCTGCTGTT ACCGTTGCAG AAGGTGTATA CGCACAGCCT TTAATCAAAG CGATATACGA

721 GGAAAAAGCC GACCTGTTAC TTGTCGGTCA AGAGACACGT TTCCGTCTCG CAAAATACCT

781 CTCCCTCATC AACGAGGACA AGAATCGTTT TGGTGTAGGT CTGGATTTCT CCGCCTATGA

841 CACTTTTCCT GTTCAAGATT TAATCCGCGA TGCATTTGCC ATCATGAAAC AAAACTTAGC

901 TTTCGGAACC TACTGGGATC CGGAAAATGG CAACGTTACC GCTGGACACG ATGATCTCCG

961 CAATTTCGAG CGAGTTCGTG CAAGAGCAGA AAAAGGATAT GACAATATCA TGGAATATTT

1021 TATCCATACC CCTTTGATTC TACCTAATGG ACGCATCATC CGCAAACATC ATGGCGTGCC

1081 ATCAGGATCA CATTTTACAA ACCTGGTCGA TTCGATTGTC AACCGATTAC TTCAAAAGAC

1141 ATTTGGATTG TACACTGAGC GATCGATTCG TGATCTCCGC ACGAATGGCG ATGACTCAGC

1201 TTTCACTGTT ACTGATACTT ACGTCGGCAA TATTCTCGAG GATGCATCTG CATTCTTCAA

1261 GAAGTGGTAC ATGACAATCA AACCAGAAAA GAGCGTGGTT GCTGGAACTC CAGCAGACAT

1321 GCATATATCT GGGACAACTT GGGAAGCTCT TCGTCCAACA CGAACCACCC AGGATTGGCT

1381 CAAGATGGCA TTGTATCCAT CAACGTATGT AAGAGACGCA AACATGTCGT TTCAACGCCT

1441 TCTCGGCATG GGAATAGCAG GCGGCTTTTA CGATGCAAAG TACTGCACTT TCTTTGAGTA

1501 CTTTCAAACT GGATACGATT GTCAACATGG TCCGAACCTT CTTTCGTGGA AGCGATTACG

1561 ATGGTTAGAA CCTGTCTTCG GCCTGAACGA CCTCCCGAAG AGTTACAAAC AGAAAAGAGC

1621 GTGGTTGCCA AGATACGTTC GCTTCTCTGG GCTCCTTAGC CCACGAGGTA CACTGAAAGC

1681 CAGTAGCTAC ATTCTCTCGT AGCGACCGT

>dsRNA2-ZD1.22-10-9

1 TTGTTCTGAA AGTCTATTGG TACGACTGCG ATTTAGTAAG AACATCTGAA CCCTTCACCA

61 ACTTCACAAA TTATCTGTTT ACATTGTGAT TGTTGAAGAC AGAAACATGG CTCCTGAGTT

121 TAAACCTGGC TGCTTCGAAC CCGTTATTAC GGTTTCCATG CGTGGAGACA GTAATACCCG

181 TGCCTTTCAT GGCGACTCCC AGGGTTTGTT TGATGGTTGT CTTGATGCTT TCAAGATTGC

241 CTTCTCTTCT TCCGCTGCAG GTGGTTCCAA TACCGAATAC ACCCGCGCAG GCATGACCAG

301 TATCTCCAAG GTTATGGCCC AATACCTTTG GGCTAGACAG TTGTCTAACC AACTCGCTCA

361 CTTTGGATTC AATGACAGGT ACCGCAAGGA AATTGCTACC GACTTTCCGC TACCTGAGCC

421 AATCGTCAAA CTCTTCAATT GCTACGGACA CGTTGAGCAT GAAGAGGTCA AGTACACCCA

481 GCTCGATCTA GAGCGTGAAT ACGCCTGGGC ACTCGTTAAC TTAGCCTGGA TGACACGTGA

541 TCTTGACTGG AATGACGAAA ATCCAACTGT GCATGACACA TTTAATGGTT GGACTACTCG

601 CCTGAACCTC AACTCAACAG GTGAGATACA AGTGGATTAC ACCGAACCCG TTCGGATGTA

661 TGTCCTGCGC TTGGTTGCGC ATATCAATGC CTTAGATGTC GATCCCGAAT ACAGGCTCCG

721 CCTGATTAAA AACATCATCG ATGTCTCCAC AGATCGTGAC CTCAACACGA TGCTCCGATG

781 GCTCCGCAAT CAGCCAAATA TTGGTAACCT CCCGCCCAGA CAAAATACCG GAAGGATAGA

841 AGCATTTGAG ATGGTCTTGC GTCAAGCCTT TCCAAATGAC GTCCCTAACT TTCGAGATGG

901 CAGGATTGGA CCCAGGGTTA TGAACCCTGT AATTCATAAG GTGACTGAAG GACTCCGAAA

961 TGTTGTTGAA GAATTGTTCA TCAACATGGC TATTGTCCAG ATGCCACGTT ATGAAAAGGG

1021 CTCGTTGGCT CAACTTTCCG AAACCACCGA TGACCTTACC TTCTCCCAGT TTCCTCTTTC

1081 ACTGGCTGAC CTTACCGTCA GTGCAGCGTT CAAAGTGGGT AGAAATCTGC GCCGCTTCCT

1141 TCGCGCATCC CCAGAACAGG TTGGTTCTTC ATTGCGAAGT GACCTAATCC GAAAATCTGT

1201 TCGTCGTCGA GCGTAATCTC TGATTATGCA CCTTTTAACA ACAGCTTCAC GACAAGTCTT

1261 TAAGCTATGA CAAATGGTTA AAGTTGCCAC GAGTTGTTAT GTAACGAAAG GTCGGACGTA

1321 GTGCTATTCG TACGCACTAC TGGCTCGCCG GTCGAGGACA AAACGTTACT CTCTTTCACT

1381 CCCTT

>dsRNA3-ZD1.22-10-9

1 AAAAGCTATT GGTACGCTTG CGATCCAGCC TGAACATCTG AACCTTCGAT CTAACCTCGA

61 AATACTGCAG CCAATACCAC CAGTATATTT TCCCTCCGCA TTGGCGAGAG ATGCATGTTG

121 ATTGCAAATT AGGTGATGGG ACCGTTGTCA CTATCAGTGA CAGATTCCCG AAGCGATGGA

181 AAAAGTTCCG AGAGAACGCC CTTTTTGCGG CTCTTTTGGT ACACTTGTTT CCCTTCGTGG

241 AAGAGGCCCT GGACGAGACG ATGTACTACG CGTTTATGTC CGATACCGAA AAGCTTAAAT

301 TATGCGTTTC GACCCTTGCC GCCAAACTCG TTTACCAACG ATTTGGACAT GTTCACTCTG

361 ACTTCTTCGA TTCTTCTTAT GGTCACGATC TGCACTTTAA TGTGTATGAA GAGAACGGAG

421 ATTACGACTT CTTTTGCGAG TGGATGTCAC GTCCAATTCT ACGCGAGTTT TCTGTGTCAA

481 TGGATGCAGC AAAAACCCTT TTTGACTTCC TGAGTTCCGC CGAAATCATT AAGGAGGAGG

541 AGCCAGAACC AATTCCACAG GAACCGATGG ACCCTTTGGA ACAATTGGTT GACAATACAC

601 CGGAAATAGC CTTTCCGCCA ATGATTCGTG TCTACGACAC GACCGAATGG CTATGTCCGA

661 TGCCTGAATG CGGGTTATGC GCATTCATCC GAACCTTTAC GAAGTTTACG CCTGACGATC

721 TTCGTAAGCA TCCCCCCAGA GATGGATGCC TTTTGGAGCG CCCACCAGAG TATCCTCTAG

781 GCTCTATTTT CTCATCTCTG CTATCACGCC CTGGCAGGAT CAAACACCCA GGACAAGCTT

841 TTTGTAGGAT AGCCGAAGCA ACTGTCTCGA ACAGATCCCT TTCTCATCGC ATGTTTGCTT

901 ATCAACATGG TGATGCTGAT GACGACTCAC CGTCTGATTC ATCATCGCTT GGGTAACTGC

961 GACTTAGGTT AGGAGTCTTG TTCTAACATA AAATTTACAA AAACACAAAA ACATATAAAA

1021 ACTTATAAAA CACCTAGATC ATTTCGTGAT TTAGTACAAA ACATTTGATT TTTGAACCCA

1081 GTACGCCGAA AGACGGAACT GTGAGTGTTG TGCTCACAGG GACTAGTCGG TCGATGTGGC

1141 GTTTAACAGA CAAAACTTAA TA
