# Supplementary material for: A Satellite dsRNA Attenuates the Induction of Helper Virus-Mediated Symptoms in Aspergillus flavus
Source: Front Microbiol. 2022 May 31;13:895844. doi: 10.3389/fmicb.2022.895844 (PMC9195127; doi:10.3389/fmicb.2022.895844)
Supplement: Supplementary file 3 [file Table_3.DOCX]

Table S2 qRT-PCR primers used in this study

| Primer name | Sequence from 5' to 3' | GeneID |
| --- | --- | --- |
| 7915878-F | TGATGCCAACCCATGCTGTC | 7915878 |
| 7915878-R | TCTCCCTGGTACAACGCCAA |  |
| 7910363-F | GCAAGCCACCATTACCGACA | 7910363 |
| 7910363-R | TCACCACGCAAGTGTGTTCC |  |
| 7920749-F | CACGGGCGAAATCGAAGTGA | 7920749 |
| 7920749-R | CCGCACGCAATGTGATCCTA |  |
| 7909819-F | AGTCCTTATTCGGCCCTGCT | 7909819 |
| 7909819-R | ATCTTGAACGGTGGGCATGG |  |
| 7911302-F | TCTCCGCTATGCTCGGTTGA | 7911302 |
| 7911302-R | AAAGCCGAAACCTGACCACG |  |
| 7922249-F | GTCGAGCCCTACCACGTTTG | 7922249 |
| 7922249-R | ATACGGACTCGGCACTTCCA |  |
| 7911577-F | GTTCGCTGTCCAACCTCAGC | 7911577 |
| 7911577-R | CGCGGAAGTAGGCAGTTTGT |  |
| 7919800-F | AAAGGCAAAGCCGAAGCAGA | 7919800 |
| 7919800-R | GCAGACTTTCCACTGTCGGG |  |
| 7916005-F | ATGTTGCGTTGGAGGTGTGG | 7916005 |
| 7916005-R | CGGTCATTTCCGTGTCGGTT |  |
| 7913731-F | CCATGTGAAGACGGTGGACG | 7913731 |
| 7913731-R | TCCCATAGCGAGCGAGTCAA |  |
| 7915880-F | TTCCAGAACCCTGCTGGTCA | 7915880 |
| 7915880-R | ATTGCGTCCGCTCTCCTTTG |  |
| 7911449-F | CCCGAATGACGCAAATCCCA | 7911449 |
| 7911449-R | ACGGCGAATGAGACCAGAGA |  |
| 7912483-F | AATCAGCGACACCACACGAC | 7912483 |
| 7912483-R | GTCACTGGCTTCTTCCTCGC |  |
| 7919618-F | GACACGTCACCAGCAGACAG | 7919618 |
| 7919618-R | CTGTGAGGTTCGGAGTTGGC |  |
| 7916928-F | CGGTTCCGCTTTAGCCCATT | 7916928 |
| 7916928-R | TTTGACCAAGCTGTCCACGG |  |
| 7910548-F | CAACCCGCTCACGTAGACAC | 7910548 |
| 7910548-R | GCGAACTCACGGTTCCCAAA |  |
| 7912479-F | CGCCGACAAACAGGTCATCA | 7912479 |
| 7912479-R | CACCGGAAGATGCCAGTCAC |  |
| 7913745-F | AAGGCCGGGATGGAGTCTTT | 7913745 |
| 7913745-R | GCTCCTCTTTGACCTGTGCG |  |
| 7910636-F | CCACTGAACCCGCAGACATC | 7910636 |
| 7910636-R | GAATGCGGACAAGGTGGGAG |  |
| 7916677-F | CGATGACCGCATTGACGAGT | 7916677 |
| 7916677-R | TCGAGTACAAGGGAGGCTGG |  |
| 7920885-F | AGCTCAAGTCTGGCGAGCTA | 7920885 |
| 7920885-R | GCAGCTTCTTACACTCCGCA |  |
| 7916140-F | GGGCTACCACCAAAGATGGC | 7916140 |
| 7916140-R | AGGCAGCATTTGAAGTCGCA |  |
| 7916990-F | TAGTGCTGCGAGTGTTGCTG | 7916990 |
| 7916990-R | CCATGTTCCTTCGGAGCCAG |  |
| 7916222-F | GGCTCGGTGATGAGTTCGTG | 7916222 |
| 7916222-R | ATGTCCAAAGCTCCCGCTTG |  |
| 7914574-F | AACCGCAGTCTCCCAACTCT | 7914574 |
| 7914574-R | ATGGCGTGCTTGTCGTCTTT |  |
| 7913140-F | AAACAAGCCTAGCACGTCGG | 7913140 |
| 7913140-R | GAACGGGCTCCTCCTCAGAT |  |
| 7917093-F | TCAAGGCGCAGGCTATGTTG | 7917093 |
| 7917093-R | CCCGGTCTCCTGTCGTATCA |  |
| 7910472-F | GCGCTTGGCGGATGAATTTG | 7910472 |
| 7910472-R | CCACCGATTGGCTTCTTGGG |  |
| 7909165-F | CTCGCAGAGTGCTTTGGTGT | 7909165 |
| 7909165-R | TGTAGCGACTCAGCCAAGGA |  |
| 7918192-F | CACAGAAGTCGCAGACCCAC | 7918192 |
| 7918192-R | TCCCACTCAGGGTAGCATCG |  |
| 7919528-F | TGTGAAATGGCGACGGAAGG | 7919528 |
| 7919528-R | GTGGTGGTCGAGGTCGTTTG |  |
| 7912799-F | AACCAGAAAGACCGCCGAAC | 7912799 |
| 7912799-R | AAGGTTGTGTTGCGGGTTGT |  |
| 7915705-F | GCTACGCACATTGGAGCCTT | 7915705 |
| 7915705-R | CTACCTTCGGCAACACCGTC |  |
| 7915229-F | GCCACCATCTGCCTGTCTTC | 7915229 |
| 7915229-R | AGCATGTGTGACCGAGAGGA |  |
| 7913727-F | GTCGATGCGAAGAAGCCCAA | 7913727 |
| 7913727-R | TCAACGACGCTACCCATCCT |  |
| 7910298-F | ACTGAGGATGACGGTACGCA | 7910298 |
| 7910298-R | GTCGGCTTTCTTGGTCGGTT |  |
| 7917152-F | TCTGTTGTGGCATGGATCGC | 7917152 |
| 7917152-R | GCAGCAGTAGTTGGCGGATT |  |
| 7913834-F | ACAACCACGAGCCGAAATCC | 7913834 |
| 7913834-R | CCACTCTGCGACTTTCGTCC |  |
| 7914574-F | AACCGCAGTCTCCCAACTCT | 7914574 |
| 7914574-R | ATGGCGTGCTTGTCGTCTTT |  |
| 7909165-F | CTCGCAGAGTGCTTTGGTGT | 7909165 |
| 7909165-R | TGTAGCGACTCAGCCAAGGA |  |
| 7910971-F | ACTCTGCTCTCCGCATTCCT | 7910971 |
| 7910971-R | TGAGCGCAGGACGAAAGAAC |  |
| 7910626-F | AGCGGAAGCAGGGTATGACT | 7910626 |
| 7910626-R | GGGCTTTCCATCCCAAGCAA |  |
| 7910463-F | TGTGGTGGTCCCGAAACTCT | 7910463 |
| 7910463-R | ATCAGTGCAGCGCCAATCTC |  |
| 7912503-F | CCATGTTGCACCTGAGCCAT | 7912503 |
| 7912503-R | GTCCTCATCCAACGCCAAGG |  |
| Afactin-F | ACGGTGTCGTCACAAACTGG | 7910404 |
| Afactin-R | CGGTTGGACTTAGGGTTGATAG |  |
