# Supplementary figures and images for: A Satellite dsRNA Attenuates the Induction of Helper Virus-Mediated Symptoms in Aspergillus flavus
Source: Front Microbiol. 2022 May 31;13:895844. doi: 10.3389/fmicb.2022.895844 (PMC9195127; doi:10.3389/fmicb.2022.895844)

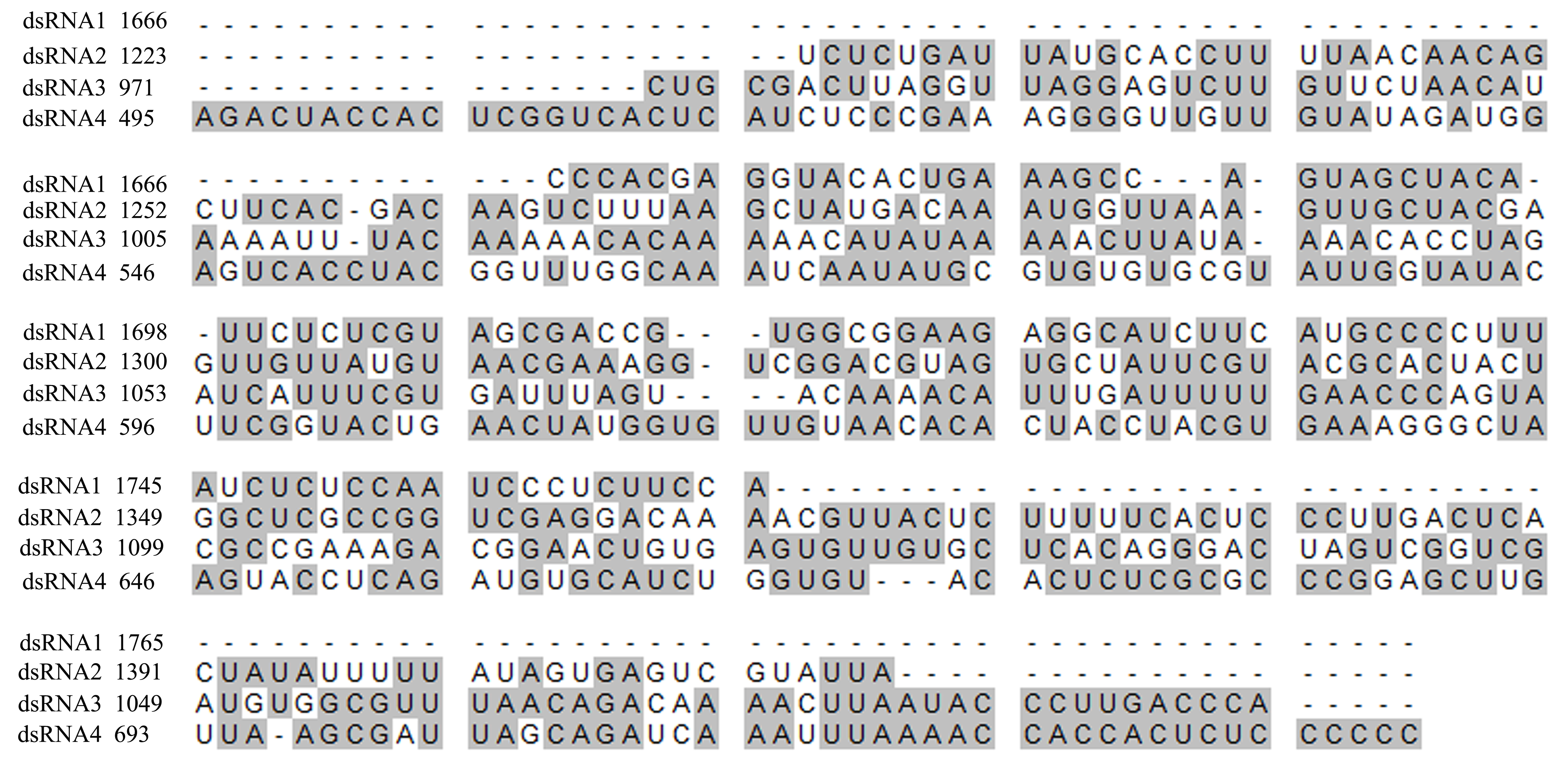

Supplement: Supplementary Figure S1 — Identity at the 3′-terminal of dsRNA1, dsRNA2, dsRNA3, and dsRNA4. Gray shading indicates where nucleotides are identical in the three segments. [file Image_1.JPEG]

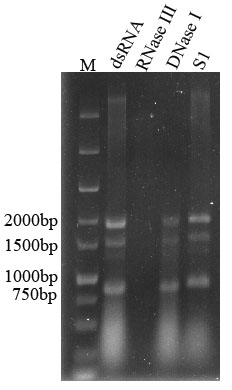

Supplement: Supplementary Figure S2 — dsRNA1, dsRNA2, dsRNA3, and dsRNA4 bands were assessed by treatments with DNase?, RNase A, or S1 nuclease. [file Image_2.JPEG]

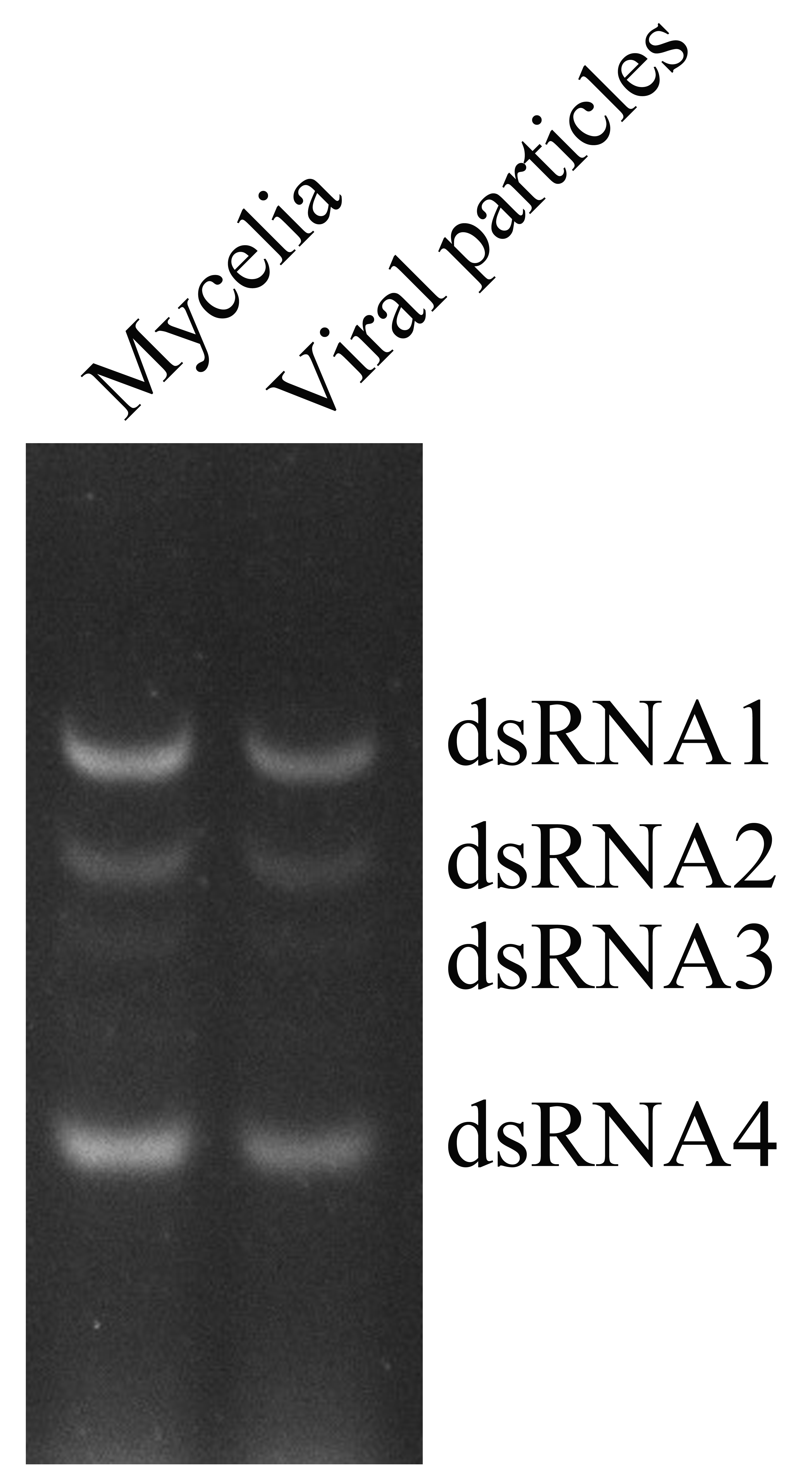

Supplement: Supplementary Figure S3 — Agarose gel electrophoresed analysis of dsRNA profiles from mycelia and viral particles. [file Image_3.JPEG]

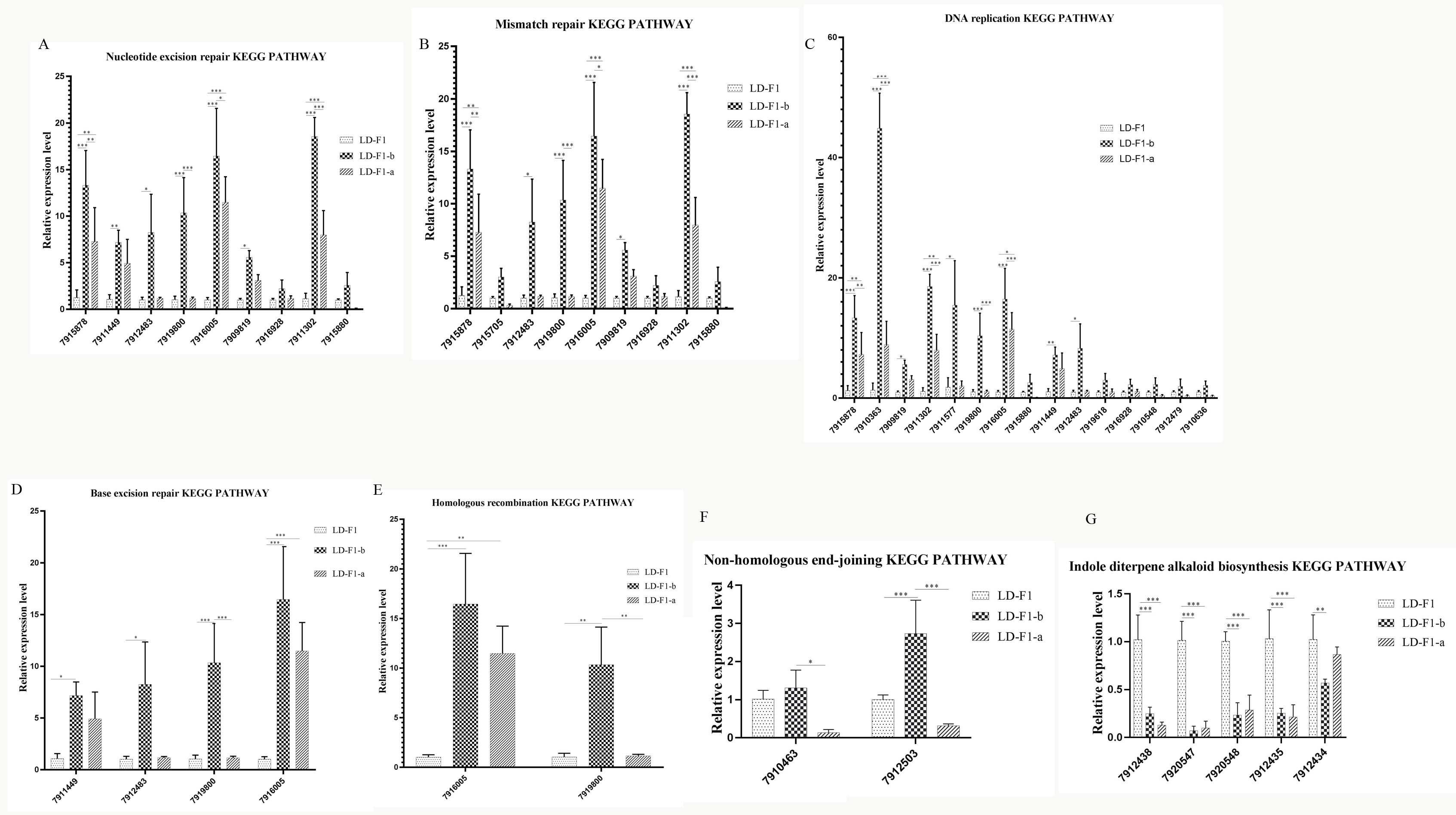

Supplement: Supplementary Figure S4 — Histological observation at 72 h post-inoculation in lung, liver, and kidney tissue infected with the virus-free isolate (LD-F1), AfPV1-infected isolate (LD-F1-b), and AfPV1- and SatRNA-infected isolate (LD-F1-a); HE staining (A) and GMS staining (B). Control experiments are comprised of non-treated mice (UTC) and saline buffer injected immunosuppressive mice (Mock). [file Image_4.JPEG]

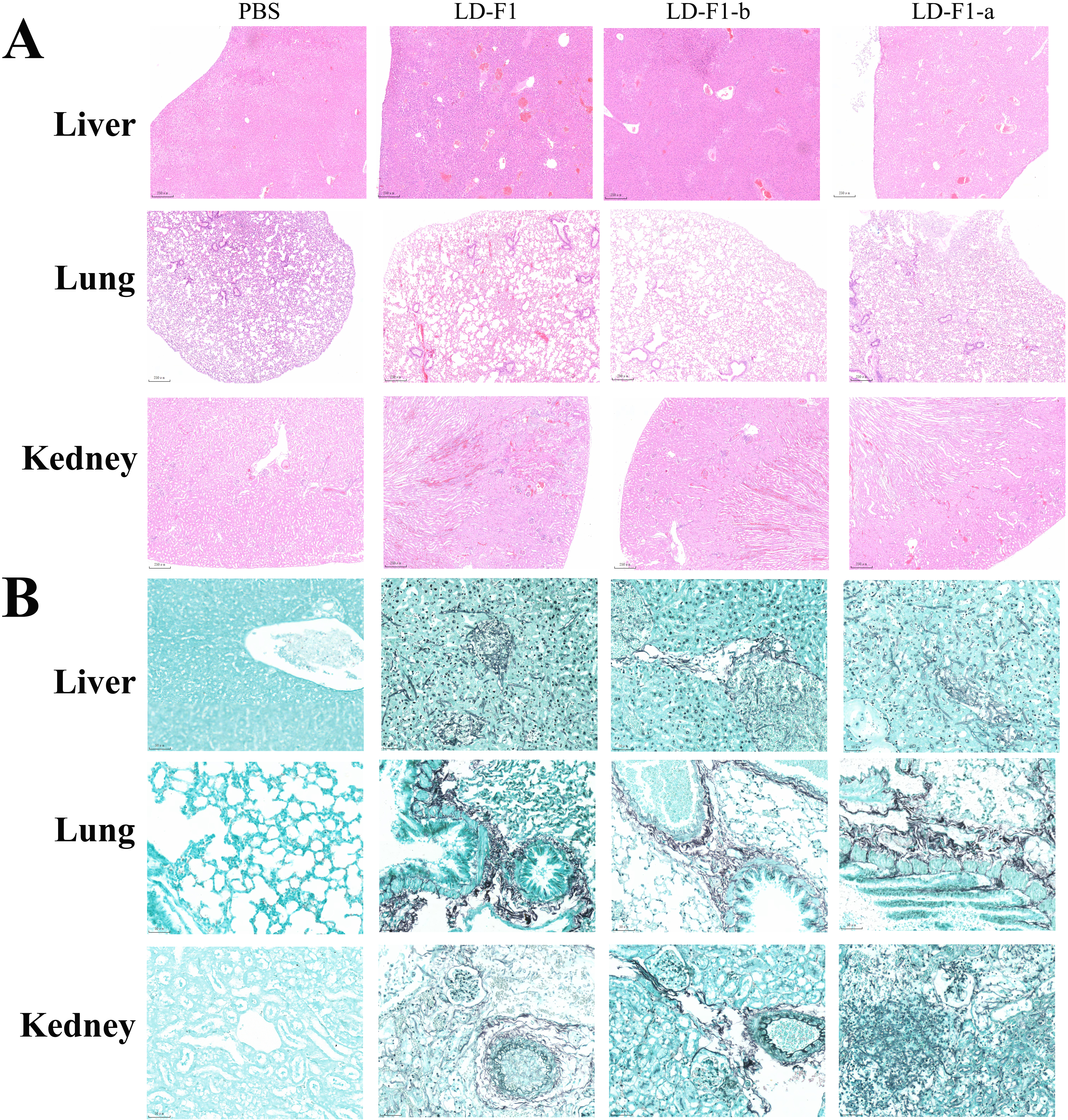

Supplement: Supplementary Figure S5 — The genes in these pathways (Non-homologous end-joining, Homologous recombination, DNA replication, Mismatch repair, Base excision repair, Nucleotide excision repair, and Indole diterpene alkaloid biosynthesis of A. flavus) were randomly selected and expression confirmed by qRT-PCR. [file Image_5.JPEG]
